# ATP‐induced conformational change of axonemal outer dynein arms revealed by cryo‐electron tomography

**DOI:** 10.15252/embj.2022112466

**Published:** 2023-04-13

**Authors:** Noemi Zimmermann, Akira Noga, Jagan Mohan Obbineni, Takashi Ishikawa

**Affiliations:** ^1^ Paul Scherrer Institut (PSI), Laboratory of Nanoscale Biology Villigen PSI Switzerland; ^2^ ETH Zurich Department of Biology Zurich Switzerland; ^3^ VIT School for Agricultural Innovations and Advanced Learning (VAIAL), VIT Vellore India

**Keywords:** axoneme, cryo‐electron tomography, dynein, ODA, power stroke, Cell Adhesion, Polarity & Cytoskeleton, Structural Biology

## Abstract

Axonemal outer dynein arm (ODA) motors generate force for ciliary beating. We analyzed three states of the ODA during the power stroke cycle using *in situ* cryo‐electron tomography, subtomogram averaging, and classification. These states of force generation depict the prepower stroke, postpower stroke, and intermediate state conformations. Comparison of these conformations to published *in vitro* atomic structures of cytoplasmic dynein, ODA, and the Shulin–ODA complex revealed differences in the orientation and position of the dynein head. Our analysis shows that in the absence of ATP, all dynein linkers interact with the AAA3/AAA4 domains, indicating that interactions with the adjacent microtubule doublet B‐tubule direct dynein orientation. For the prepower stroke conformation, there were changes in the tail that is anchored on the A‐tubule. We built models starting with available high‐resolution structures to generate a best‐fitting model structure for the *in situ* pre‐ and postpower stroke ODA conformations, thereby showing that ODA in a complex with Shulin adopts a similar conformation as the active prepower stroke ODA in the axoneme.

## Graphical abstract

Cryo‐electron tomography study of the outer dynein arm from the unicellular alga *C. reinhardtii* provides insights into previously unseen *in situ* structural features.

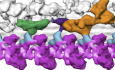

## Introduction

Motile cilia are rod‐like extensions protruding from the cell body. Their function is to provide movement to the organism itself or to the adjacent environment. They exist from unicellular eukaryotes such as *Chlamydomonas* and *Tetrahymena* to humans. In humans, motile cilia function in various tissues, such as the lung, brain, and reproductive organs, as well as sperm, and a defect causes ciliopathies such as primary ciliary dyskinesia (PCD; Hildebrandt *et al*, 2011). The beating motion of cilia takes place in the part extending out of the cell body, called the axoneme. In most species, the axoneme has a “9 + 2” structure, in which nine microtubule doublets (MTD) surround two singlet microtubules. The MTD comprises a cylindrical microtubule with 13 protofilaments (A‐tubule) and an incomplete B‐tubule with 10 protofilaments (Ma *et al*, 2019). Until now, more than 400 proteins have been identified in motile cilia (Pazour *et al*, 2005).

The driving force of ciliary motion is generated by axonemal dyneins. They are anchored on a microtubule doublet and generate force against the adjacent doublet by hydrolyzing ATP. In most species, axonemal dyneins are incorporated into two complexes—outer and inner dynein arms—which make periodic arrays with 24‐ and 96‐nm periodicity, respectively. The outer dynein arm (ODA) has either two or three heavy chains (HC), stacking on the doublet (Goodenough, 1982; Nicastro *et al*, 2006; Ishikawa *et al*, 2007; Rao *et al*, 2021; Walton *et al*, 2021), whereas eight HCs make an array in the inner dynein arm (IDA). Functionally ODA is responsible for force generation, whereas IDA has regulatory functions (Kamiya, 2002). Each HC of the axonemal dynein consists of an N‐terminal tail domain followed by the motor domain. This includes six AAA motifs (named AAA1–AAA6), with a microtubule‐binding domain (MTBD) connected to AAA4 by a coiled‐coil stalk, similar to its homolog cytoplasmic dynein. The α‐helix‐rich linker region is between the tail and AAA1 (Carter *et al*, 2011; Kon *et al*, 2011). Additionally, to the HC, there are several light and intermediate chains present in the ODA and IDA, alongside with light‐intermediate chains for the IDA. The tail complex of the ODA is formed by the N‐terminal tails of the HC (Kubo *et al*, 2021; Mali *et al*, 2021; Rao *et al*, 2021; Walton *et al*, 2021). For *Chlamydomonas*, the β and γ tails form the scaffold for the two intermediate (IC1 and IC2) and multiple light chains (LC1‐LC10, including LC7a/b). Most of the LC form the light chain tower (LC‐tower), which sits on the γ tail. Only LC1 and LC4 are associated with the γ tail at the outside of the LC tower. LC3 and the kelch domain of the α HC bind to the β‐tail (Mali *et al*, 2021). The α HC docks on the β‐tail at helical bundle 6 (Rao *et al*, 2021). The N‐terminal end of the γ and β‐tail forms the N‐terminal dimerization domain (NDD). This tail complex is anchored near the NDD on the A‐tubule by DC3 as well as DC1 and DC2.

Both axonemal and cytoplasmic dyneins make a power stroke (PS) with respect to the microtubule, releasing γ phosphate after ATP hydrolysis (Johnson, 1983, 1985). A single‐particle cryo‐EM (SPA) study of the motor domain from axonemal dynein demonstrated the swing of the linker during the power stroke (Roberts *et al*, 2009). The same conformational change of the motor domain from cytoplasmic dynein has also been studied at atomic resolution (Carter *et al*, 2011; Schmidt *et al*, 2015). The linker interacts with the interface of AAA4 in the post‐PS conformation and AAA2 in the pre‐PS conformation (Carter *et al*, 2011; Kon *et al*, 2012; Schmidt *et al*, 2015), which is consistent with single‐particle cryo‐EM work of isolated axonemal dynein (Roberts *et al*, 2009) and cryo‐EM/ET work of ODA in the axoneme (Ueno *et al*, 2014; Lin & Nicastro, 2018; Kubo *et al*, 2021; Walton *et al*, 2021) at intermediate resolution. Recent high‐resolution SPA has shown that there are two different linker conformations present in the axonemal ODA of *Tetrahymena* (Kubo *et al*, 2021; Rao *et al*, 2021) for the post‐PS structure, the so‐called Post‐1 and Post‐2 conformations. While in the Post‐1 conformation the linker goes over AAA4, in the Post‐2 conformation the linker interacts with the interface of AAA3 and AAA4. The Post‐2 conformation was thus far only observed for DHY4 and DHY5 (β‐ and α‐HC analog in *Chlamydomonas*). Two microtubule‐binding states (MTBS) have been described by Rao *et al* (2021) MTBS1 (Post‐1, Post‐1, Post‐2 conformation) and MTBS2 (Post‐1, Post‐2, Post‐2 conformation). These models are summarized in Appendix Table S1.

Recent work revealed that during reciliation of the ODA in *Tetrahymena*, the ODA is in a closed conformation, where the DHY3 (γ‐HC ortholog) is folded onto the other two HC (Mali *et al*, 2021). This conformation is held together and inhibited by Shulin. In this Shulin–ODA complex, the dynein linker adopts a pre‐PS conformation. The stalks are curved and interact with each other close to their MTBDs. Once Shulin dissociates from the Shulin–ODA complex in the axoneme, the ODA is no longer inhibited, and opens up and binds to the A‐tubule. For this, the DHY3 undergoes a 90° rotation together with DIC2 (IC1 analog) and the LC tower (Mali *et al*, 2021; Rao *et al*, 2021).

The interaction between the MTBD and the B‐tubule has been studied by cryo‐EM (Carter *et al*, 2008; Redwine *et al*, 2012; Rao *et al*, 2021). The MTBDs bind on the protofilaments of the B‐tubule, between α‐ and β‐tubulins, under the strong binding condition of the post‐PS state (Carter *et al*, 2008; Redwine *et al*, 2012), but changes its binding angle in the pre‐PS state (Redwine *et al*, 2012). The stalk, protruding from AAA4, tilts toward the proximal direction (minus end of MTD) both in the pre‐ and post‐PS conditions (Ueno *et al*, 2008, 2014; Movassagh *et al*, 2010; Lin *et al*, 2014). This indicates that the sliding motion between two adjacent doublets is caused by dynein, which is anchored on the A‐tubule, dragging the adjacent B‐tubule (winch model; Ueno *et al*, 2008; Movassagh *et al*, 2010), rather than rotating to push the B‐tubule (rotation model). Due to a lack of resolution in the imaging of dyneins generating force against the MT, the precise conformational change of the axonemal dynein head during a PS is not known.

Single‐particle cryo‐EM successfully revealed a 3D conformational change of the motor domain of isolated cytoplasmic and axonemal dyneins. However, this method so far lacked information on the ODA in cilia with and without ATP. This knowledge is necessary to explain both how the ODA generate force in cilia and how ciliary beating occurs. To this end, we visualized the entire ODA structure in cilia with and without ATP by cryo‐electron tomography (cryo‐ET). To describe the structural change, we fitted published atomic structures of ODA, either anchored on the A‐tubule but isolated from the B‐tubule (Kubo *et al*, 2021; Walton *et al*, 2021) or connected to the B‐tubule but not anchored on the A‐tubule (Rao *et al*, 2021), to the structure revealed by cryo‐ET. First, we described how the ODA structure in the axoneme differs from those structures analyzed by single‐particle cryo‐EM. Next, we used molecular fitting to build in a model of the *in situ* post‐PS ODA structure, as well as a model of the native pre‐PS ODA structure. Based on these models at ~ 3 nm resolution, we could discuss the mechanism of force generation of an ODA and its interaction with the adjacent B‐tubule.

## Results

Classification of subtomograms from cryo‐ET of *Chlamydomonas* axoneme in the presence of ATP (Appendix Table S2) led to six different classes. One of these classes represents the MTD‐1, which was previously shown to lack ODA (Bui *et al*, 2012), while another class represents the pre‐PS conformation, as shown later. The other four classes likely represent conformations after post‐PS and before pre‐PS, judging from the position of the dynein head being more proximal than in the post‐PS conformation and more distal than in the pre‐PS conformation. We therefore call these ODA conformations intermediate states. No purely post‐PS state with all three HCs in a post‐PS conformation was found in these subaverages. We thus have obtained an apo state, depicting the post‐PS conformation from a nucleotide‐free dataset.

### Post‐PS structure and fitting models

Recently, several structures of ODA in the post‐PS were solved by single‐particle cryo‐EM (PDB‐7KZM, 7MOQ, 7K58, and 7K5B; Appendix Fig S1 and Appendix Table S1). These structures are from specimens where the ODAs are either natively anchored on the A‐tubule (7KZM and 7MOQ) or isolated and reconstituted on the adjacent B‐tubule (7K58 and 7K5B). All these structures are without nucleotides. While the structure of 7KZM stems from *Chlamydomonas*, the remaining three are from *Tetrahymena*.

7KZM has modeled the three dynein heads, but it is lacking a C‐terminal part of each HC tail and the stalks. It has both IC1, IC2, LC2, LC4, LC6, LC7a and b, LC8, LC9, and LC10 modeled, as well as a complete ODA‐DC complex and A‐tubule (Walton *et al*, 2021). 7MOQ has the complete Dyh3 and Dhy4 HC without the stalks, DIC2 and DIC3 and all light chains except LC1 and LC5 modeled (Kubo *et al*, 2021). 7K58 and 7K5B have all three HC including the stalks on the B‐tubule modeled. All light and intermediate chains are present, and only the ODA‐DC complex is missing (Rao *et al*, 2021).

**Figure 1:**
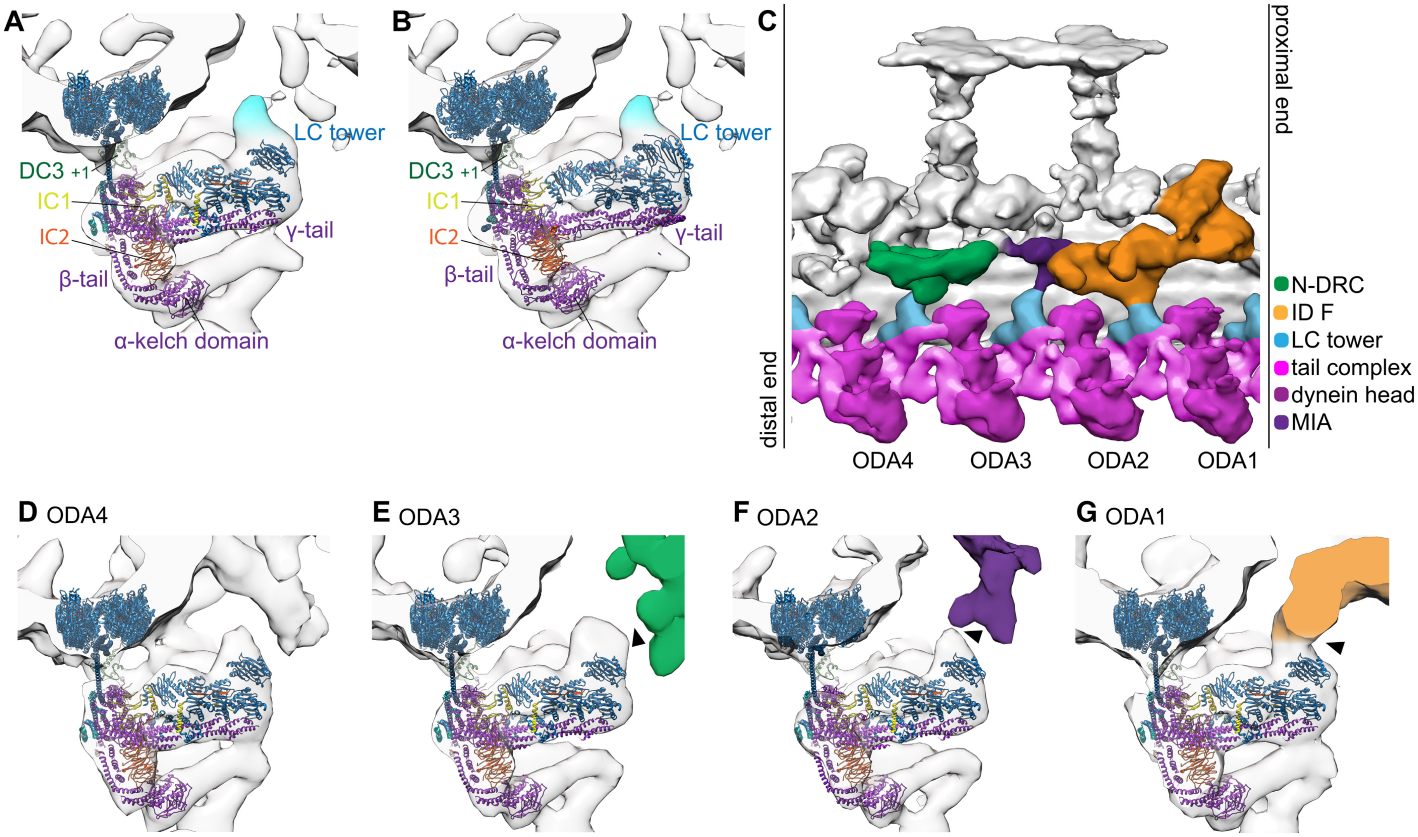
Tail ODA structure by subtomogram averaging from cryo‐ET of Chlamydomonas cilia A, BLC tower of 24‐nm subtomogram average of Chlamydomonas, with the model PDB‐7KZM (A) and PDB‐7MOQ (B) fitted. Fitting was done to maximize the cross‐correlation between the whole ODA structure and the tomographic map. An unassigned density above the LC tower is highlighted in light blue.C96‐nm unit from post‐PS Chlamydomonas by subtomogram averaging.D–GEach LC tower of the 96‐nm average viewed from the end‐on view. The LC tower connects once every 96 nm to the IC/LC complex of inner dynein f (G, “ODA1”) and to the MIA complex (F, “ODA2”). The LC tower also comes close to the N‐DRC (green) but does not seem to form a strong interaction with it (E, “ODA3”). All outer dynein‐to‐inner dynein connections are indicated by black arrows.

**Figure 2:**
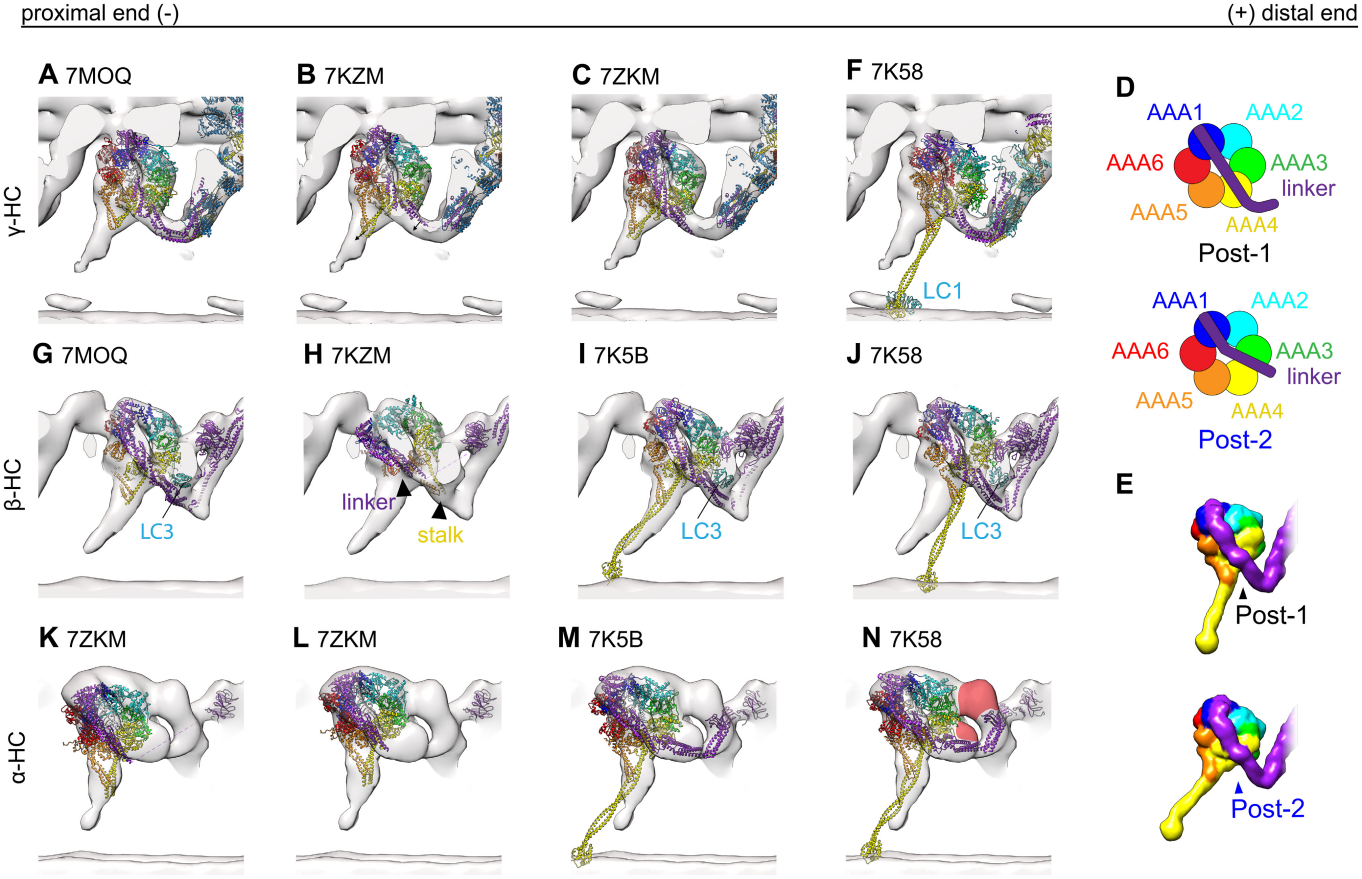
Comparison of models single‐particle analysis post‐PS ODA and our cyro‐ET subtomogram averaging map A–Cγ‐dynein map shown with different A‐tubule anchored PDB structures. (A) 7MOQ γ‐head rigid body fitted with the whole ODA. The stalk and linker are slightly pointing out of the density. With a slight rotation and translation of the stalk and head of the γ‐dynein, the fit becomes better. However, the linker is not in agreement with the density map. (B) 7KZM the same rotation and translation as for 7MOQ could improve the fit between structure and map. (C) After rigid body fitting of the dynein head individually, the 7KZM structure fits better.D, ESchematic representation of Post‐1 and Post‐2 linker conformations (Rao *et al*, 2021). (D) 20 Å map calculated from a Post‐1 (black) and Post‐2 (blue) conformation.FMTBS1 and 2 have the γ‐dynein in post‐1 conformation, which is not in agreement without tomographic map.G–Jβ‐dynein map shown with different PDB models. (G) 7MOQ β‐dynein, where Kubo *et al* proposed a new linker conformation similar to post‐2 of Rao *et al* (2021). In (H) the linker and the stalk of the 7ZKM β‐dynein point into the same direction, indicated by black arrowheads. (I) MTBS2 where the dynein in post‐2 conformation fits well with our map. (J) MTBS1 where the β‐dynein is in post‐1 conformation.K–NPost‐PS α‐HC tomographic map with the PDB models. The α‐HC of the 7ZKM model is lightly outside the tomographic density when the whole ODA is rigid body fitted (K). The fit can be improved once the α‐HC head is fitted individually from the rest (L). Both MTBS1 and MTBS2 have the α‐head in a Post‐2 conformation (M and N). In N the unassigned density found for all α‐HCs is highlighted in red.

**Figure 3:**
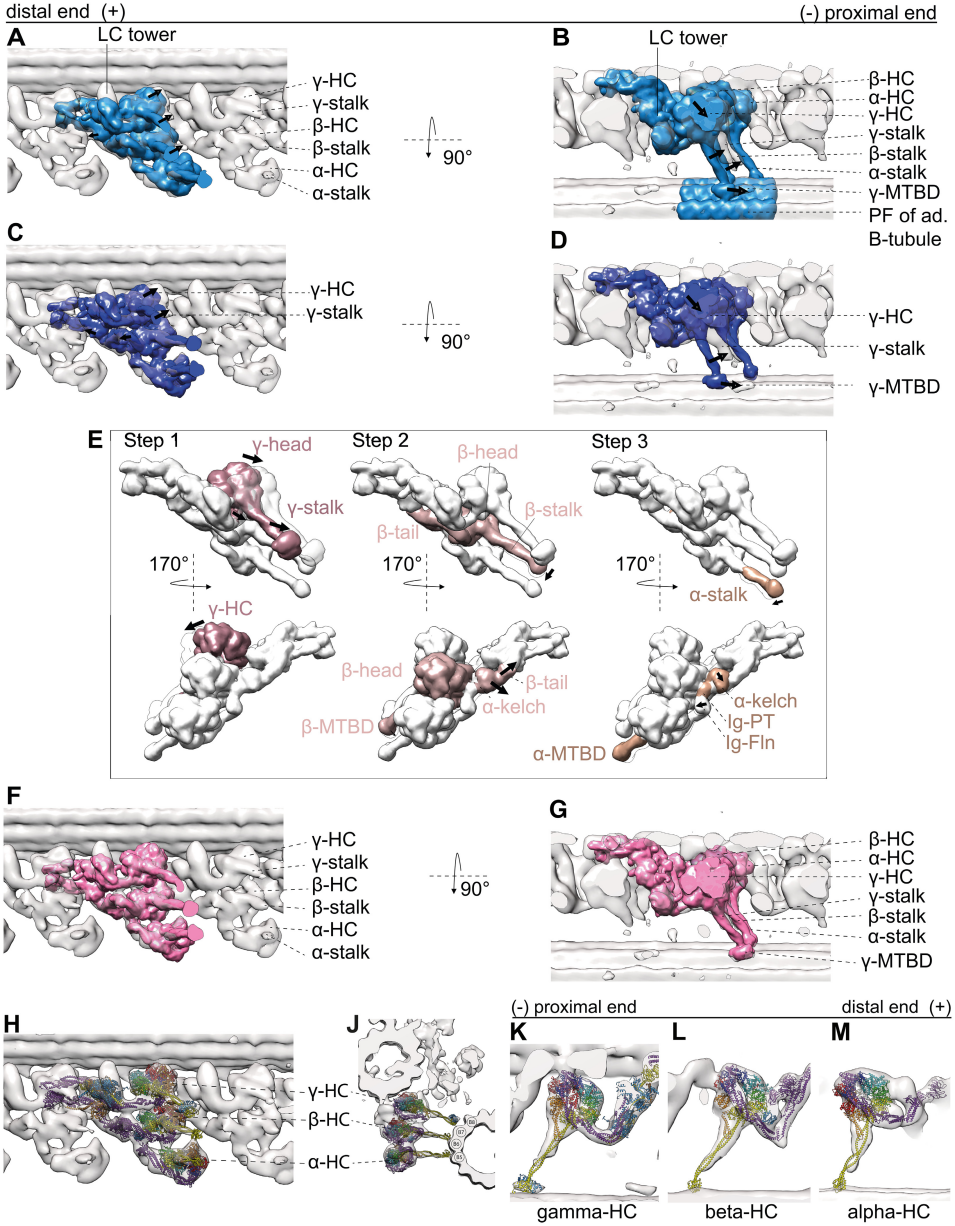
20 Å resolution map calculated from the MTBS1 and MTBS2 (PDB‐7K5B and PDB‐7K58) model and fitted to our tomographic map to highlight the difference of stalk B‐tubule binding between the *in situ* and *in vitro* structures A, BRigid body fit of the whole MTBS1 map (Walton *et al*, 2021). There is discrepancy at the dynein heads, including the stalks and the MTBDs (indicated by the arrows) and the tomographic map.C, DRigid body fit of the whole MTBS2, there biggest difference between the map and the model is in the γ dynein head, stalk and its MTBD. There are also some smaller discrepancies for the β and α HC.ESteps necessary to fit the MTBS2 model to the tomographic map. Step 1: The γ head needs to shift. Changing the linker position from a Post‐1 conformation to a Post‐2 conformation. Step 2: The β‐HC, from helical bundle 5 to the β‐dynein head, shifts slightly. The two binding partners LC3 and the α kelch domain move with the β‐tail. Step 3: The stalk and MTBD of the α‐HC shifts. The α kelch domain moves closer to the N‐terminal end of the β‐tail.F, G20 Å resolution map calculated real‐space‐refined model (MTBS3) after the three steps.H–MThe MTBS3 model fitted into the tomographic map. While H and J show the overview of the ODA, K–M show the individual HC with the MTBS3 fitted to the γ (K) β (L) α (M) HC, respectively. All three HC are modeled in a Post‐2 conformation.

We compared these models with our *Chlamydomonas* cryo‐ET subtomogram averaging map without additional nucleotide (Figs 1 and 2) and furthermore attempted to fit real‐space‐refined structures of the ODA by remodeling the PDB models to our map (Figs 2 and 3; discussed later).

### LC tower

In all four SPA models, the LC tower, fitted as a rigid body, does not completely fill out our tomographic density: in each 24‐nm repetitive unit, there is an additional density above the LC tower (light blue in Fig 1A and B). This density does not connect to the inner dyneins via ODA4 (Fig 1C and D) and comes close but does not connect to the N‐DRC (“ODA3” in Fig 1C and E). On the contrary, the density connects to the IC/LC of inner dynein f and the MIA complex (Yamamoto *et al*, 2013; “ODA2” and “ODA1” in Fig 1C, F and G).

### γ‐HC

The structures generated by reconstitution of ODA on the MTD (MTBS1 (PDB‐7K58) and MTBS2 (PDB‐7K5B; Rao *et al*, 2021)) were fitted to our tomographic map via rigid body fitting. Both models (PDB‐7KZM; Walton *et al*, 2021 and PBD‐7MOQ; Kubo *et al*, 2021), in which the ODA is anchored on the A‐tubule, were fitted based on the DC3 and the PF (all cross‐correlation values are in Appendix Table S1). With this alignment, the γ‐HC of the PDB‐7MOQ model is slightly (approx. 2 nm) more distal than in the PDB‐7KZM, and 7KZM fits better to the *in situ* structure (Fig 2A and B). The buttress/stalk and linker position (AAA4) for the γ dynein are very similar between the two A‐tubule anchored models, but contact the B‐tubule at a 1–2 nm more distal than the tomographic map suggests (Fig 2A and B). Individual fitting of the γ head of PDB‐7KZM results in a rotation and translation of the head with respect to the tail, increasing the fit between the map and the model (Fig 2B vs C, Movie EV1). However, the position of the linker is less in agreement with the tomographic map. If this ever so slight head rotation would pull the C‐terminal end of the tail closer to the A‐tubule, which is indeed suggested by the tomographic map, the γ dynein linker would be in a Post‐2 conformation, where the linker spans over AAA3/AAA4 (Kubo *et al*, 2021; Rao *et al*, 2021). A schematic representation of the Post‐1 and Post‐2 conformation can be seen in Fig 2D and E. Consistently, the B‐tubule bound gamma dynein head (PDB‐7K58 and 7K5B both Post‐1) also needs to undergo a similar rotation to fit the dynein head, stalk and MTBD into the tomographic map (Fig 2F).

### β‐HC

The 7MOQ β‐HC structure of Kubo *et al* overlays perfectly with our tomographic map (Fig 2G). Their proposed linker position over AAA3/AAA4 (Post‐2) and LC3 positions also align with our tomographic map (Fig 2G). Walton *et al* found that the stalk of the β‐HC points distally in the majority of their particles. So far, no such structure was observed in our subtomogram averages (Fig 2H). The dynein head of MTBS2, being in the Post‐2 conformation, confirms that the tomographic map is indeed in a Post‐2 conformation (Fig 2I), as the fit of MTBS2 is much better than that of MTBS1 (Fig 2J).

### α‐HC

When we fitted the SPA models with the third HC, either α of *Chlamydomonas* or DHY5/γ of *Tetrahymena* (PDB‐7KZM, 7K58, and 7K5B), we found an additional density next to the α dynein head visible in the tomographic map (Fig 2K–N). This suggests that in the *in situ* structure there is an additional protein density present between AAA2 and AAA3 of the α dynein as well as the Ig‐Fln of the α tail (Fig 2N, red colored), which has fallen off or became smeared out due to flexibility during single‐particle analysis.

It might be the density of one of two known binding partners of the α HC, Lis1, or LC5 (King, 2016). In *Chlamydomonas* Lis1 binds as a monomer (Rompolas *et al*, 2012, 1). Although Lis1 could fit into the unassigned density, this density binds to AAA2/3 instead of the previously stated interaction for Lis, AAA4/5 (Htet *et al*, 2020).

The other discrepancies between the SPA models and the tomographic map of the α HC are most likely due to displacement. The α dynein head of 7KMQ appears too proximal and close to the adjacent B‐tubule (Fig 2K). Individually fitting the α‐head of PDB‐7KZM results in a good fit between the SPA model and the subtomogram average (Fig 2L). This individual fitting changes the distance between the kelch‐like β propeller and the dynein head, moving them closer together. Therefore, the distance between the kelch‐like β propeller and the head structure is larger in single‐particle analysis than in cryo‐ET (Fig 2L). The comparison of the tomographic map and the PDB‐7K58 and PDB‐7K5B models shows that the linker of the α‐HC is in a Post‐2 conformation in the tomographic map (Fig 2M and N).

### Inter‐ and intra‐ODA connections

Inter‐ and intra‐ODA interactions appear to be as described by Walton *et al* and Kubo *et al* for the γ and β‐HC. The γ‐HC connects to the proximal γ‐tail (helical bundle 3) close to the DC3 and via the linker to the β‐HC within the same ODA (Appendix Fig S2A and B). The β‐HC connects to the proximal tail structure at the region of IC2 (Appendix Fig S2A and B) and also to the proximal NDD (Appendix Fig S2A and B). An additional connection is observed from the tomographic maps between the AAA3 α HC and the β linker (Appendix Fig S2A and B).

### MTBS1, MTBS2, and MTBS3

So far two different MTBS have been described for the post‐PS ODA (Rao *et al*, 2021), where MTBS1 has a Post‐1:Post‐1:Post‐2 conformation and MTBS2 has a Post‐1:Post‐2:Post‐2 conformation for the γ:β:α HC, respectively. As mentioned above, the tomographic map suggests that the γ linker is in a Post‐2 conformation, which was observed in neither MTBS1 nor MTBS2. *In situ* all three linkers appear to be in a Post‐2 conformation. MTBS1 (Fig 3A and B) fits worse than the MTBS2 model (Fig 3C and D; Appendix Table S3), which has the β and α homolog linker in a Post‐2 conformation. Additionally, since the distal end of the ODA is not anchored on the A‐tubule, the C‐terminal tail of both structures and the LC towers appear too proximal. To optimize the fitting of the MTBS2 to the cryo‐ET map, we performed rigid body fitting and real‐space refinement of individual parts of the structure by Coot (Fig 3E). This optimization included three steps. Step 1 is the translation and rotation of the γ dynein head including the MTBD. This shifts the MTBD by 8 nm, which corresponds to one step on the adjacent B‐tubule. The linker of the γ‐HC changes from a Post‐1 conformation to a Post‐2 conformation (Movie EV2). Step 2 includes a 1 nm backward rotation of the β‐HC from helical bundle 5 up to the β‐dynein head. In addition to the β‐HC, this also moves LC3 and the α kelch domain so that they fit better into the tomographic map. Step 3 focuses on the α HC, with the kelch domain moving 1 nm closer to helical bundle 5 of the β‐HC. In addition, the stalk of the α‐HC adapts to a more straight conformation by a 1 nm distal movement of the MTBD. The resulting MTBS3 structure is shown in Fig 3F–M. Through this remodeling, the cross‐correlation score between the 20 Å filtered MTBS2 and MTBS3 structures and the cryo‐ET map increased from 0.6797 to 0.8402 (Appendix Table S3, Movie EV3).

### Pre‐ and intermediate structures

**Figure 4:**
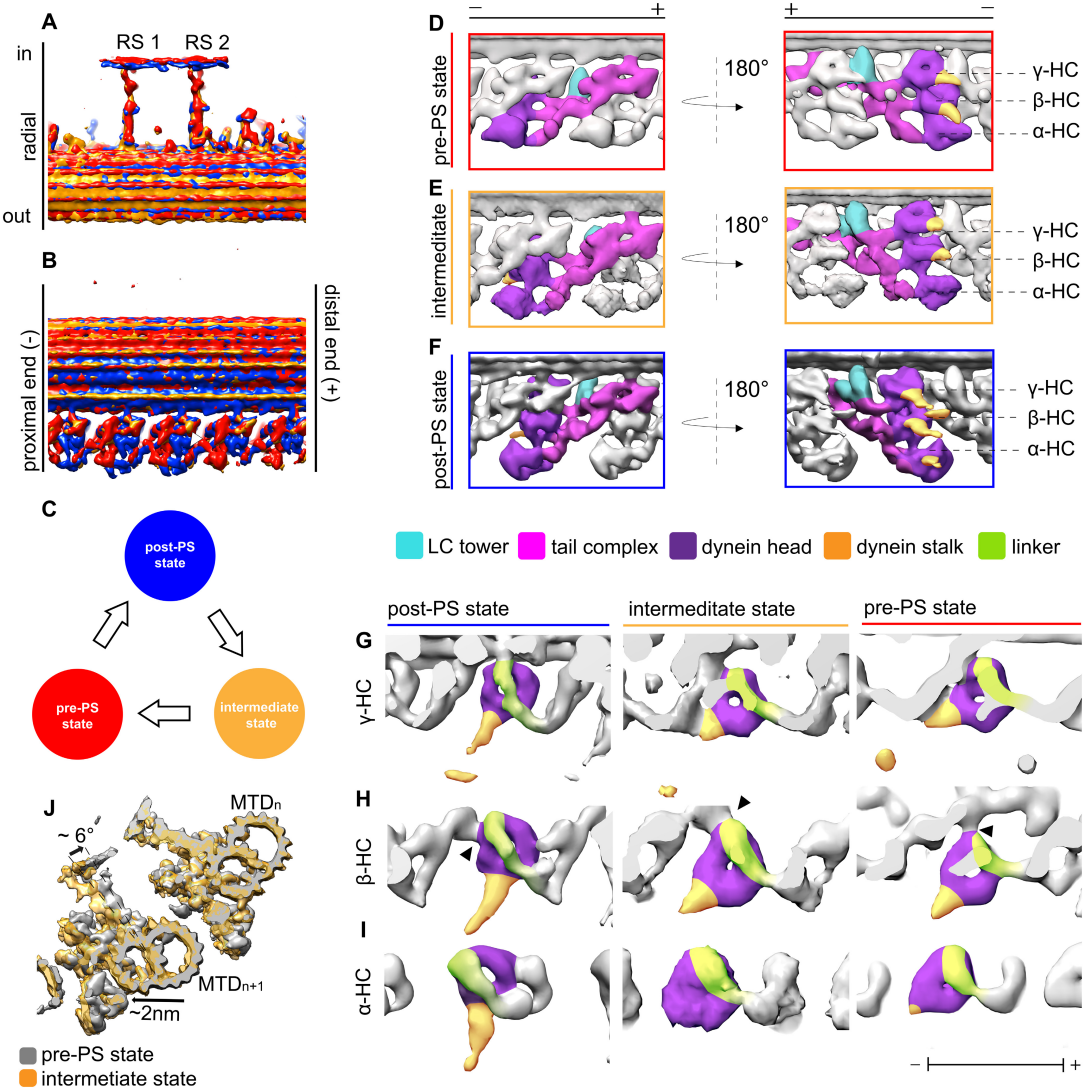
Structural changes of ODA between different states A, BOverlay of the 96‐nm array of the three states (post‐PS, pre‐PS, and intermediate state). The structures are aligned based on the RS. With this, the DC3 (indicated by white arrowhead) are also aligned.CModel of the three states. The intermediate state is after the post and before the pre‐PS states.D–FThe dynein heads move toward the proximal direction in the intermediate (E) and pre‐PS state (D) compared with the post‐PS state (F). Also the LC tower moves in the pre‐PS state compared with the post‐PS.G–Iγ (G), β (H) and α (I) HC in the post‐PS, intermediate and pre‐PS conformation. Linker position for all the HC and states is indicated in green, while the stalk and the AAA ring are shown in yellow and purple, respectively. The black arrowhead in H indicates the interaction of the β‐dynein head to the proximally located IC2, which is consistent in all three states.JOverlay of the intermediate (orange) and pre‐PS (gray) map. Shown are two MTDs. The position of the adjacent B‐tubule changes between the two maps. In the intermediate conformation, the next MTD (“*n* + 1”) moves approximately 2‐nm away compared with the pre‐state (indicated by black arrow). Furthermore, the whole *n* + 1 MTD rotates by around 5°.

In the presence of ATP, RELION 3D classification detected six conformations of ODA in the wild‐type *Chlamydomonas* cilia (Fig 4). One resembles the previously known pre‐PS state since (i) the positions of the heads are ~8 nm proximal to the post‐PS structure, similarly to the ODA structure with ADP and vanadate (Movassagh *et al*, 2010) and (ii) the orientation of the linker is the same as cytoplasmic dynein with ADP and vanadate (Schmidt *et al*, 2015). The other structure with all the three HC in the same conformation, which we define as an intermediate conformation, shares numerous features with the pre‐PS, such as a dynein head position closer to pre‐ than post‐PS structures and linkers in a pre‐PS‐like conformation. In addition to these purely pre‐ and intermediate conformations, three classes were detected which had the γ and β‐HC in either a post‐PS or intermediate conformation. We assigned these classes to be subclasses of the intermediate state, as they are probably conformations of the recovery stroke (discussed later).

Alignment of the different tomographic maps was done based on the RS, which led the DC3 to overlap (Fig 4A and B). We propose that the ODA recovery stroke starts at the post‐PS state, transitions via the intermediate states and ends primed for the power stroke in the pre‐PS conformation (schematics Fig 4C). During this recovery stroke, the dynein heads move from their distal post‐PS conformation, via their intermediate position to their most proximal pre‐PS conformation (Fig 4D–F). With this movement, the linker changes from a straight post‐PS conformation to a 90°‐bent pre‐PS conformation in the intermediate and pre‐PS structure (more detail below; Fig 4G and H). For the pre‐PS state, the stalks are more strongly tilted parallel to the adjacent B‐tubule than for the intermediate structure (Fig 4D and E). Although classification was done based on only the central ODA, there are more global changes found outside of the ODA. By overlaying (Fig 4J), it becomes apparent that the distance and angle between the two adjacent MTDs changed, whereby the angle between two adjacent MTDs in the intermediate conformation is wider than for the pre‐PS conformation (Fig 4J). Based on the position of the subtomograms in the tomogram, the angles between two adjacent MTDs were calculated to be 40.2 ± 6.3 degrees (Median ± interquartile range [IQR]) for the post‐PS conformation (*n* = 854), 39.8 ± 10.1 degrees for the intermediate (*n* = 223) and 35.6 ± 11.19 degrees for the pre‐PS conformation (*n* = 91). In addition to this slight rotation of the adjacent MTD, in the intermediate conformation the distance between the ODA and MTD based on the subtomogram average is approx. 2 nm longer compared with the pre‐PS conformation (movement indicated in Fig 4J). The distance based on two neighboring subtomograms in the tomogram was calculated to be 60 ± 15 nm (median ± IQR) for the pre‐PS (*n* = 91), 66 ± 9 nm for the post‐PS conformation (*n* = 854), and 67 ± 8 nm for the intermediate states (*n* = 223) of the ODA.

**Figure 5:**
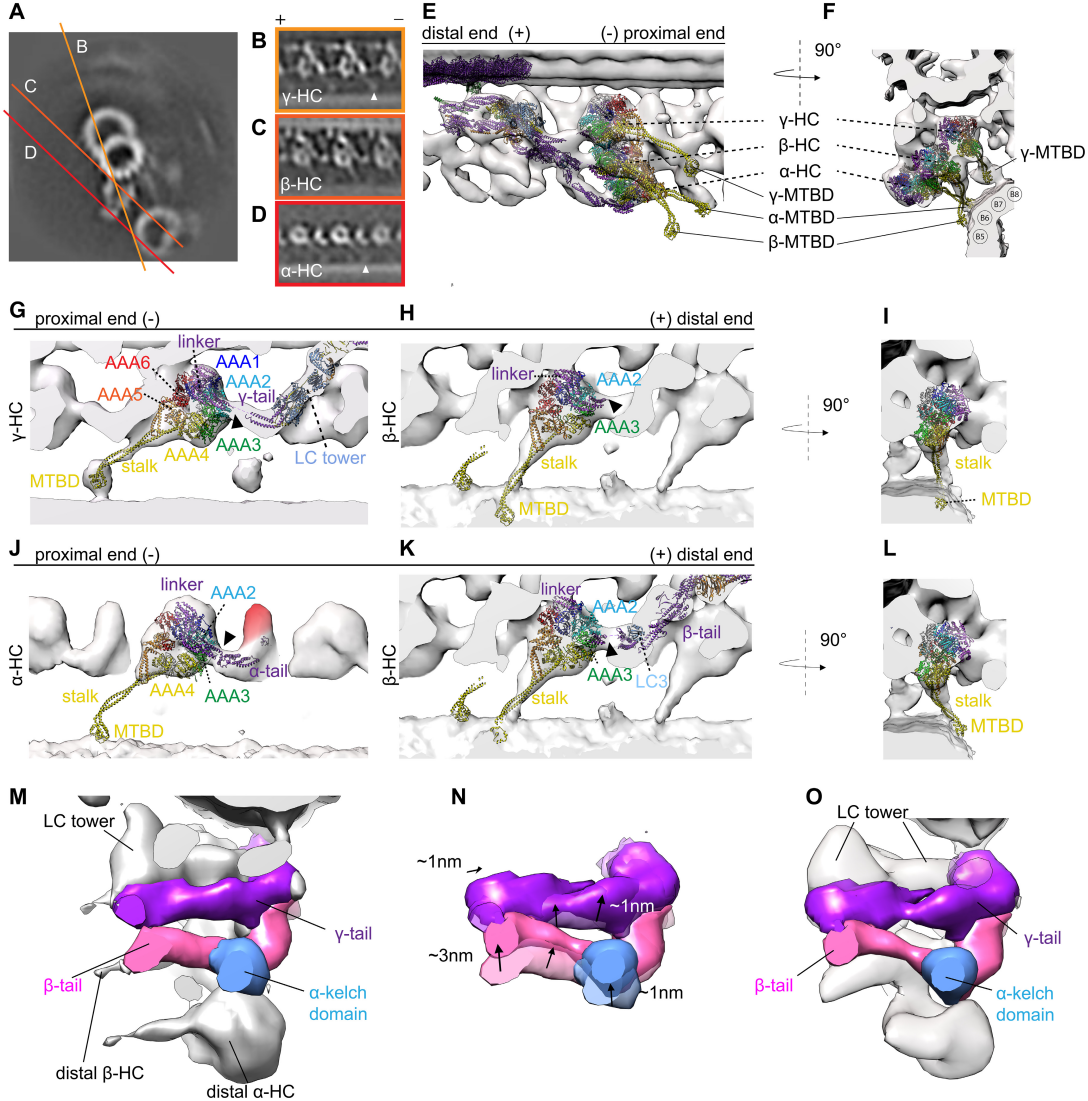
Pre‐PS conformation of the ODA ACross section of one MTD in the pre‐PS conformation.B–DTomographic slices for the γ (B), β (C) and α HC (D), respectively. The MTBD of the γ and α dynein is indicated with a white arrowhead.E, FTwo views of the overall structure of ODA in the pre‐PS conformation with the pre‐PS model (γ PDB‐4RH7, β & α PDB‐6ZYW and modified tail of PDB‐7MOQ) fitted.G–LEnlarged view of individual dyneins in surface‐rendered representations with the pre‐PS model juxtaposed. The linker‐to‐tail connections are indicated by black arrowheads. The cytoplasmic pre‐PS dynein PDB‐4RH7 (Schmidt *et al*, 2015) shows that the γ linker is in a bend conformation and that the MTBD is contacting the adjacent B‐tubule. The stalk is visible for the β‐dynein (C, H, I, K, L). If the stalk had an extended conformation like in the cytoplasmic PDB‐4RH7 conformation, the MTBD of the β‐HC would penetrate into the adjacent B‐tubule (H, I). This is due to the dynein head position, which is too close to the adjacent B‐tubule. The β stalk rather appears to be in a bend conformation which is much more similar to the conformation adapted in the Shulin–ODA complex (Mali *et al*, 2021) (K and L). The head of the α‐Shulin–ODA (J) is in agreement to the tomographic map. The linker and the MTBD are located in tomographic density.M20 Å resolution map of the post‐PS tail complex (calculated from PDB‐7MOQ) shown in a pre‐PS tomographic map.N20 Å resolution map of the real‐space‐refined pre‐PS model overlaid on post‐PS tail to highlight the changes.O20 Å resolution map of the real‐space‐refined pre‐PS model fitted in the pre‐PS tomographic map.

In the pre‐PS conformation, the dynein heads have moved ~ 8 nm and the γ MTBD ~ 13 nm proximally compared with the post‐PS conformation (Fig 4D–F). In this pre‐PS conformation, the linker is in a 90°‐bent conformation, which is similar to the single‐particle cryo‐EM analysis of cytoplasmic and axonemal dyneins heads in the presence of ADP‐VO_4_ (Roberts *et al*, 2009; Schmidt *et al*, 2015), while the stalks are visible and point proximally toward the adjacent B‐tubule (green and yellow in Fig 4G–I, respectively). All these observations suggest that this is indeed the pre‐PS conformation. From the subtomogram average (Fig 5A–D) as well as the surface‐rendered model (Fig 5E–L), one can see that all three stalks are approaching the adjacent B‐tubule.

The γ stalk of the prestructure is in an extended conformation, attaching with its MTBD to the adjacent B‐tubule between PF 7 and 8 (Fig 5B, F, and G). The stalk of the β‐dynein appears to be in a curved conformation, similar to the conformation it adapts in the Shulin–ODA complex (Mali *et al*, 2021; Fig 5C, E, F, K, and L). If this stalk were straight, the MTBD of β would be embedded in the B‐tubule (Fig 5H and I). The MTBD of β is not clearly visible. A weak density between the PF5‐6 on the adjacent B‐tubule suggests that the MTBD of the α HC binds to the B‐tubule with the α stalk in the usual extended conformation (Fig 5D–F and J). With these stalk conformations, the MTBD of the β‐dynein is not only more distal than the extended stalk of the α dynein (Fig 5E) but also more radially outwards (Fig 5F). In this case, the α and β stalks would have to cross during a PS cycle (Movie EV4).

With the combination of rigid body fitting of the crystallographically solved cytoplasmic dynein in the pre‐PS and the recently solved dynein HC in the Shulin–ODA complex in which ATP binds, we were able to generate a real‐space‐refined model for the pre‐PS state, which agrees with our tomographic maps (Fig 5F, K, and L). The linker position of the cytoplasmic dynein coincides with the tomographic map of the prestructure for the γ‐ and β‐HC (Fig 4G–I). For these two HC, the linker conformation spanning over AAA2 is the same as in the Shulin–ODA. The linker for the α dynein adopts a slightly different conformation from the cytoplasmic dynein. While the linker still has a 90° bend, it runs a little closer to AAA3, as seen in the Shulin–ODA complex. For our pre‐PS map, the tail‐linker connection can be seen for all three dynein HC (Fig 5G, H, and J). The volume between the linker and the tail in the pre‐PS and the intermediate structures corresponds to the number of missing residues. However, for the α dynein the empty volume between the linker and the kelch‐like β propeller is too large to correspond only to the α linker. In the post‐PS state, this unknown density has connections to the α tail and the AAA2/3 (Fig 2N). In the pre‐PS map, the unassigned density appears only next to the Ig‐Fln of the α tail, suggesting that during the PS cycle the connection to the dynein head is lost (Fig 5J).

Due to the weak tomographic density around the MTBDs, the MTBDs of the input PBDs were not real‐space refined. The α‐ and β‐HC stalk from Shulin–ODA, but not cytoplasmic dynein, fit well to our density map, as both the map and the model adopt a curved conformation. Only γ‐HC shows a resemblance between cytoplasmic dynein and our map. The stalk of the γ‐dynein is in the straight conformation (Fig 5G).

The tail complex of the post‐PS ODA (PDB‐7MOQ) fits overall to our pre‐PS maps, meaning that the conformational change inside the tail complex is relatively small compared with the dynein head change. The docking complex and the N‐terminal region of the γ and β‐tail show high similarity to the post‐PS form in the pre‐PS state. In the pre‐PS conformation, the proximal region from helical bundle 4 for the γ tail and 5 for the β‐tail up to the dynein heads deviates from the post‐PS conformation (Fig 5M). To fit to our cryo‐ET map, the γ and β‐tail should move together with the LC‐tower around 1 nm proximally and 1 nm closer to the A‐tubule (Fig 5N and O). The β‐tail at helical bundle 7–9 moves an additional 2 nm closer to the γ tail (Fig 5N). If this movement would not occur, the dynein head in the pre‐PS conformation would sterically clash with the post‐PS LC tower position.

**Figure 6:**
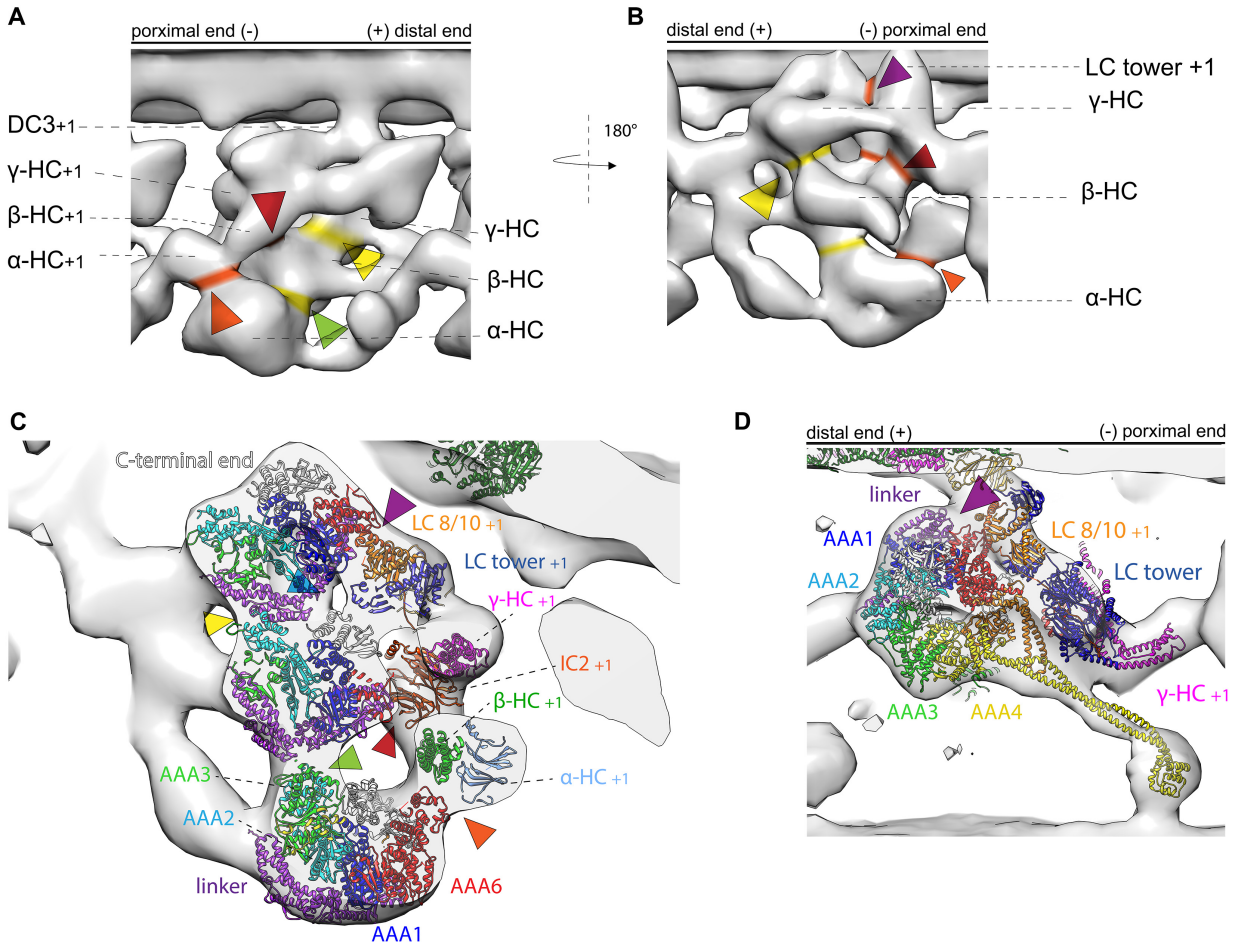
Intra‐ and inter‐ODA connection in the pre‐PS conformation A, BSurface‐rendered model of pre‐PS conformation. Intra‐ODA connections between the three HC are highlighted in yellow, and inter‐ODA connections are highlighted in orange.CSection through the three dynein chains. The real‐space‐refined model of the pre‐PS has been fitted into the cryo‐ET map to highlight which parts of the ODA are involved in the interaction. The γ linker connects to the AAA2 from the β‐HC (yellow arrowhead). The β linker connects to the AAA2/AAA3 of the α HC (green arrowhead). AAA6 (purple arrowhead) and the stalk from the γ‐HC (pink arrowhead), as well as the C terminus of the β‐HC (light blue arrowhead), interact with the LC tower. The AAA6 from the β‐HC interacts with the proximal IC2 (red arrowhead). The AAA6 from the α HC interacts with the β‐tail of the proximal ODA (orange arrowhead).DView of the γ‐HC with the PDBX‐4RH7 highlighting the interaction between the γ‐HC and the proximal LC tower. AAA6 interacts at LC8/10 level (purple arrowhead) and the stalk interacts with LC6/8 (pink arrowhead).

Rao *et al* have described the tail‐to‐head interactions occurring in the post‐PS of *Tetrahymena* ODA and suggested that these interactions need to be broken for the PS to occur. We aimed to look at the intra‐ and inter‐ODA interactions in the ODAs in the pre‐PS conformation. The following densities connecting the dynein heads to other dynein heads within the same ODA (intra‐ODA connection, indicated in Fig 6A and B, yellow) and to the tail from the proximal ODA (inter‐ODA connections, indicated in Fig 6A and B, orange) were observed. In the pre‐PS conformation, the γ‐HC density connects the LC tower via its AAA5/AAA6 site to LC8/LC10 (Fig 6B–D, indicated by the purple arrowhead). The stalk of the γ‐HC is in close proximity to the LC tower at the region of LC8/6 (Fig 6D, pink arrowhead). The intra‐ODA density between the γ‐HC and β‐HC corresponds to the γ linker connecting to AAA2 of the β‐HC (Fig 6A–C yellow arrowhead). There is a weak connection of the β‐HC to the LC‐tower in the region of LC8/10 (Fig 6C, indicated by a blue arrowhead). A more dominant connection of the C‐terminal end of the β linker is established to IC2 (Fig 6A–C, red arrowhead). In the case of the α‐HC, the N‐terminal region of the β‐linker is involved in a connection to the AAA2/AAA3 region (Fig 6A and C, green arrowhead). The α‐HC shows a density connection to the proximal β‐tail in the area of the kelch domain of the proximal α HC (Fig 6A–C, orange arrowhead).

**Figure 7:**
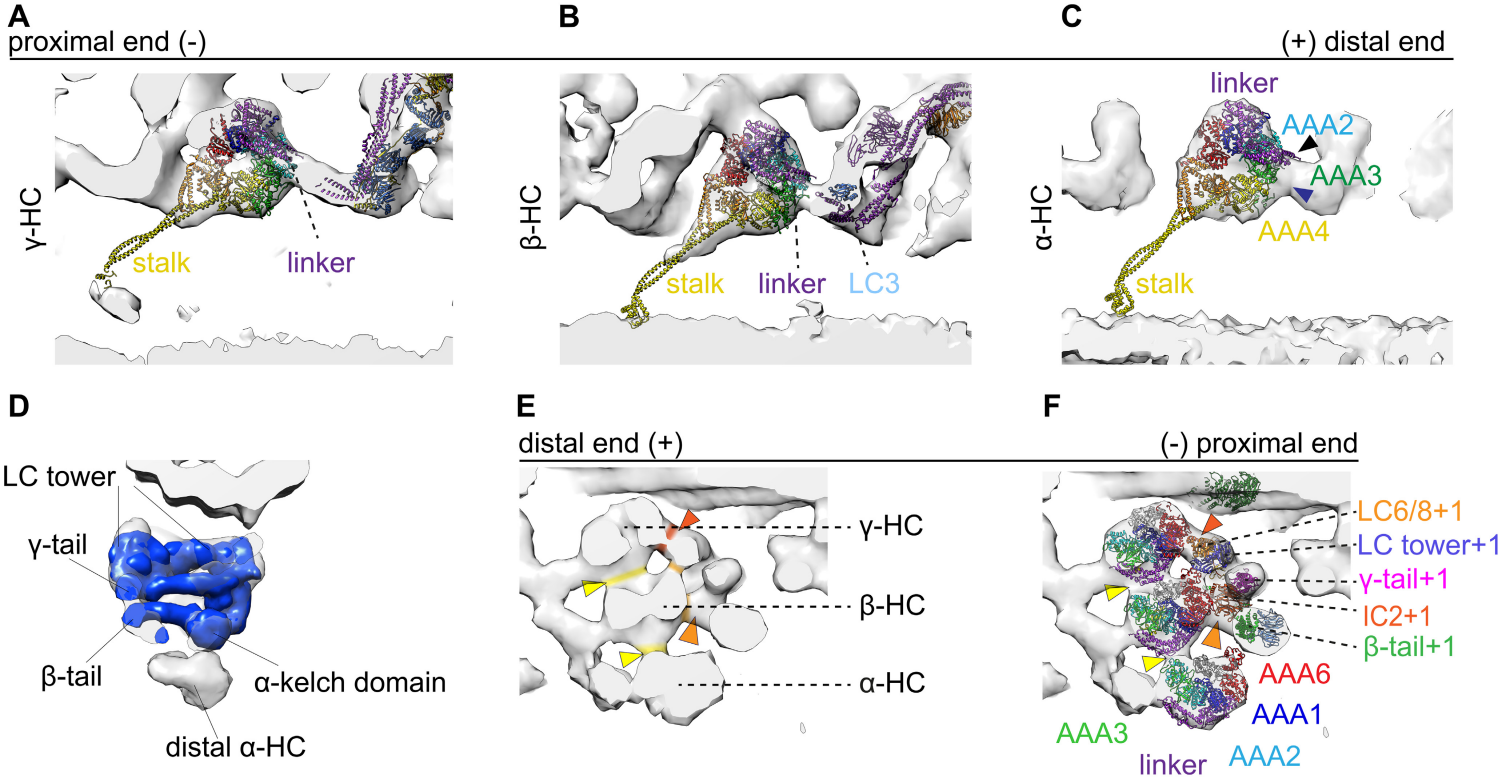
Intermediate conformation A–CSurface render maps of the γ (A) β (B) α HC (C) are shown with the cytoplasmic pre‐PS dynein PDB‐4RH7 (Schmidt *et al*, 2015) rigid body fitted. The tail complex from PBD‐7MOQ is fitted as a rigid body. The bend linker of the PDB structure is in agreement with the γ‐ (A) and β‐HC (B). For α‐HC (C), the linker (purple) does adapt an intermediate conformation, whereas the linker spans over AAA3 (bottom blue arrowhead) instead of going over AAA2 (top black arrowhead).DThere are no major deviations between the tail structure of the postmodel and the intermediate conformation tomographic map.EThe intra‐ODA connection between the β‐head and theγ‐linker is highlighted in yellow. The inter‐ODA connections of the β‐dynein head to the IC2 and the connection of the γ head to the LC tower are indicated in orange.FPDB structures fitted to the same view as in (E), highlighting which proteins are involved in the tail‐to‐head interactions. The highlighted regions from I are marked with arrows in E. The linker of the γ and β‐HC is interacting with the AAA3 of the β and α HC, respectively (yellow arrows). AAA6 of the γ‐HC interacts with the proximal LC tower (red arrow). The AAA6 of the β‐HC interacts with the IC2 of the proximal ODA (light orange arrow).

We found one intermediate ODA conformation with all three dynein HC in the same conformation and three structures with mixed dynein conformation. The dynein heads of the all‐intermediate state are located between the post‐PS and the pre‐PS conformation (Fig 4E). The stalks of the dynein heads in this intermediate conformation are partially visible (Fig 7A–C). The base of the stalks close to the dynein head is pointing proximally. There is no sign of stalks crossing, unlikely the pre‐PS. The linker of this intermediate conformation appears similar to the pre‐PS linker conformation discussed above (Fig 4G–I). For the α HC, the linker in the intermediate conformation seems to go over AAA3 (Fig 7C indicated by black arrow), between the pre‐like (AAA2/AAA3) and post‐like (AAA3/AAA4) linker conformation. The unassigned density of the α HC is detached from the α head and connected to the α tail, as was seen for the pre‐PS conformation (Fig 2J). Since the distance to the adjacent B‐tubule is the same between the post‐PS and the intermediate conformations, but the linker appears to have already changed to a pre‐like conformation, the stalks can be in a straight conformation without protruding into the adjacent B‐tubule (Fig 7A–C). At the current resolution, the tail region resembles the post‐PS conformation (Fig 7D). The tail‐to‐head interactions in the all‐intermediate conformation resemble the tail‐to‐head interaction of the pre‐PS conformation (Fig 7E and F). The γ dynein head has detached from the NDD and now forms an interaction with the LC tower of the proximally located ODA (Fig 7E and F, orange indication). The γ linker interacts with the β‐head, and the β‐linker with the α head, respectively (Fig 7E and F, yellow arrowhead). Similar to the pre‐ and post‐PS conformation, the β‐dynein contacts the tail structure at IC2 (Fig 7E and F, light orange indication).

**Figure 8:**
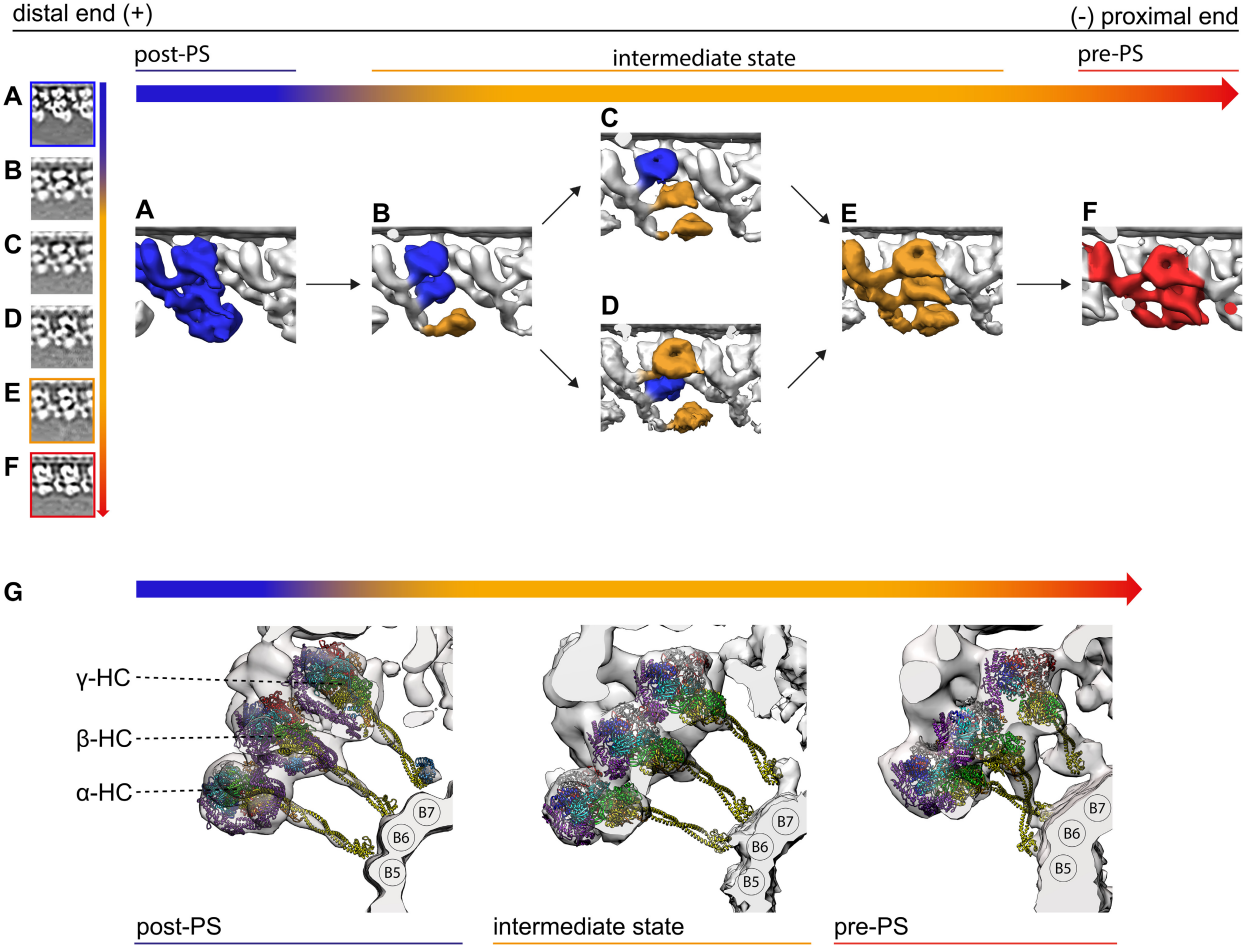
Recovery stroke of ODA A–FAll conformation of the ODA arranged in a proposed sequence during the recovery stroke. For all states, the ODA is shown in a tomographic cross section and as a surface‐rendered map. (A) Apo ODA in an all post‐PS conformation from the nucleotide‐free dataset. (B) The αHC moved to an intermediate state and appears blurred. (C) The β‐HC and αHC are in an intermediate conformation. (D) The γ and αHC are in an intermediate conformation. (E) All three HC are in an intermediate conformation. (F) All three dynein HC adopt the prepower stroke conformation.GStalk orientation of the post‐PS, intermediate, and pre‐PS state. The tomographic maps suggest that in the post‐PS as well as the intermediate state the stalks are in a parallel, straight conformation. In the pre‐PS state, however, the map suggests that the stalks of the α‐ and β‐HC cross over.

Relative to the post‐PS conformation (Fig 8A) the mixed intermediate conformations have the γ and β head in either a post‐PS‐like or an intermediate‐like conformation (Fig 8B–D and H–J). The α‐HC in all these conformations is smeared out but adopts an intermediate‐like conformation (Fig 8E). One of these intermediate subclasses has both the γ and β‐HC in a post‐PS‐like conformation (Fig 8B), and the other had either β or γ in a post‐PS‐like and the other HC in the intermediate conformation, respectively (Fig 8C vs D). No mixture with the pre‐PS conformation (Fig 8F) was found.

## Discussion

In this study, we have determined the *in situ* structure of ODA of *Chlamydomonas* cilia by cryo‐ET and demonstrated differences from the *in vitro* structures in the post‐PS conformation by single‐particle analysis. This difference could come from the difference in the method of preparation—whereas cryo‐ET represents the more native state, SPA requires more purification steps. SPA can thereby lead to higher resolution for but also distort the native conformation.

The minimum energy conformation when only one end is anchored on either the A‐tubule or the B‐tubule can differ from that of the native conformation, where the ODA structure is restricted by both A‐ and B‐tubules. Moreover, none of the SPA structures solved had all the components present. PDB‐7KZM and PDB‐7MOQ lack the adjacent B‐tubule, and PDB‐7K5B and PDB‐7K58 lack the docking complex and are assembled *in vitro* on MTDs. Removal of such structures, which might exert some force through binding on the ODA, could change the overall ODA structure. In the post‐PS, the MTBD is attached to the adjacent B‐tubule. Without this binding partner, the stalk is highly flexible and can take up conformations which would not be possible in the native environment, such as the β stalk pointing distally as found in PBD‐7KZM. In contrast to all the SPA structures, we found all three HC in a Post‐2 conformation as defined by Kubo *et al* (2021) and Rao *et al* (2021). We refer to this structure as MTBS3. There might be an unknown factor to bias equilibrium between Post‐1 and Post‐2 toward Post‐2 dominance. Furthermore, an additional density appearing in the *Chlamydomonas* ODA in cryo‐ET (Figs 2N and 5J). Such smaller component can fall off during purification for SPA, be smeared out during the averaging process due to flexibility or be results from species differences. PDB‐7K5B, PDB‐7K58, PDB‐7MOQ, and PDB‐6ZYW are from *Tetrahymena*. Based on sequence alignment of *Tetrahymena* and *Chlamydomonas* dynein HC α (Uniprot ID|17M6H4| and Uniprot ID |A0A2K3DV97|, respectively), we found ~ 340 amino acids missing at the dynein neck region of *Tetrahymena*, which could correspond to parts of the unassigned density found in *Chlamydomonas*. However, there is no density difference between these models and our structure from *Chlamydomonas*, which presents these amino acids.

Upon the addition of ATP, we found six ODA conformation by 3D classification with RELION. This could be due to the higher nucleotide concentration compared with the previous work (Movassagh *et al*, 2010). Our intermediate conformations lack the higher resolution information, such as the full‐length stalk. We speculate that the intermediate states represent transitional states in which the linker of either individual or all HC already underwent the conformational change while the stalk is still finding the right PF to bind on the adjacent B‐tubule. Our pre‐PS map resembles the previously described ATP‐VO_4_ state (Movassagh *et al*, 2010). We therefore assign this conformation to the ADP.Pi state, while the major intermediate state probably represents the ATP state.

By the cryo‐ET analysis of cilia, we demonstrated how the interaction partner, the B‐tubule, influences the ODA confirmation. This becomes apparent when we look at the different conformations during the PS cycle. The distance and angle of the neighboring MTD changed in the presence of ATP. This can be explained by the interaction of the adjacent B‐tubule with the stalks and MTBDs. In the post‐PS conformation, the stalks are upright and extended (Fig 8G), maintaining a certain distance between two MTDs. Once ATP binds and subsequently the MTBD detaches from the adjacent B‐tubule, all three dynein heads move proximally by changing their linker position from post‐ to pre‐PS conformation. The γ head breaks the tail‐to‐head interaction with the NDD and forms new connections with the LC tower in the proximal ODA. During the whole PS cycle, the interaction between β‐HC and IC2 seems to be maintained. Although the resolution is not high enough to see changes inside IC2 directly, if we assume that IC2 does not shift with respect to the rest of the tail complex, it could serve as an origin of rotation for the β‐HC. The mixed intermediate states indicate that the β and γ‐HC can bind ATP independently and move to the intermediate position separately. Once all three HC have ATP bound they adopt the intermediate conformation. To move from the intermediate to the pre‐PS conformation, ATP is hydrolyzed and the MTBD binds the adjacent B‐tubule. Differently from the model proposed by Lin *et al* (2014), in which the conformational change occurs by rotation of the dynein head and stalk toward the distal end, our data suggest that the dynein heads move even more proximal than in the intermediate conformation, pushing the tail complex of the next ODA in the same direction. By binding to the adjacent B‐tubule, the ODA can exert a force on the MTD, which is bound by DC3, thereby pulling itself 2 nm closer to the adjacent B‐tubule. Pointing of the stalk of the β‐dynein curved toward PF 5 (which was observed also in previous findings in *sea urchin sperm* flagella by Lin *et al*, 2014, Fig 8G) can introduce a torque, rotating the ODA relative to the adjacent MTD. Previously, from cytoplasmic dyneins, it was believed that the power stroke happens in one step, but this cannot be the case for the β‐dynein since the binding on PF seems to change between pre‐PS and post‐PS structures. This opens up the question of whether the β‐dynein takes on a different role or there is another state after pre‐PS and before post‐PS.

In all the three states and all the dyneins, the stalks extend in the proximal direction, which supports the winch hypothesis (Ueno *et al*, 2008). However, the stalks are tilted more toward the proximal end in the pre‐PS than the post‐PS conformation, suggesting active rotation during the PS. The dynein heads change conformations in slightly different ways. The β‐dynein rotates around and moves with IC2 (Fig 4H, the connection to IC2 is indicated with black arrowheads) while γ and α do more translational movement (Fig 4G and I). While β rotates and shifts only very little compared with the other two HC, the α head can shift more proximally than the β head, which could allow the α stalk to bind the PF more proximally than the β‐stalk (Fig 5E and F).

Recently, it was proposed that the ODA is prepared for transport to the axoneme in an inactive Shulin–ODA complex. This structure was then solved by Mali *et al* (2021). Kubo *et al* proposed how to open this Shulin–ODA complex to be in a post‐PS conformation bound to the A‐tubule. Our finding shows that the pre‐PS conformation of the α and β‐HC in the Shulin–ODA complex and those from the ODA bound to the axonemes are almost the same. The β‐HC is similar to the cytoplasmic dynein in the pre‐PS state. With this, we were able to build a real‐space‐refined pre‐ODA model. While for the precise atomic structure of the pre‐PS ODA we need to wait for further high‐resolution study, our result can give important insight into the pre‐PS conformation. It shows that the linker in the axonemal pre‐PS dynein is at 90° and that the MTBD of γ and α bind on the adjacent B‐tubule (Fig 5B, D, and G–L). It furthermore highlights that a curved stalk conformation, as seen for the pre‐PS β‐dynein, can occur natively. Apart from a global shift, and a local shift of the β‐tail closer to the γ tail in the pre‐state, our tomographic map was not resolved well enough to see structural changes in the tail complex. However, our data suggest that there is indeed some structural change happening in the tail complex during the PS cycle (Fig 5M–O).

In conclusion, we have shown there are differences between SPA and cryo‐ET results. We have built a model of the pre‐PS state (at intermediate resolution) and show that the linker, head, and stalks of the Shulin–ODA HC represent the native pre‐PS ODA conformation.

## Materials and Methods

### Cells

*Chlamydomonas* cells (CC‐125^+^ from the *Chlamydomonas* resource center) were cultured in TAP medium with a 12‐h dark and 12‐h light cycle. The cells were collected by centrifugation and deflagellated with dibucaine hydrochloride (Sigma‐Aldrich, USA) following the Witman procedure (Witman, 1986). Flagella were collected by centrifugation at 22,000 *g* for 5 min. Axonemes were demembraned by 0.5% NP‐40 treatment in 30 mM HEPES (pH 7.4), 5 mM MgSO_4_, 1 mM DTT, 1 mM EGTA, 50 mM potassium acetate buffer. The activity of the purified axonemes was verified under the Zeiss Axioskop (Germany) light microscope at 40x magnification upon the addition of 1 mM ATP.

### EM grids

R3.5/1 Holey carbon copper grids (QUANTIFOIL, Germany) were glow‐discharged under UV light. 10 nm gold beads were applied to the grids as fiducial markers. The axoneme solution was mixed with ATP (final concentration of 1 mM ATP) and brought to 0.1 mg/ml protein concentration. This activated solution was immediately applied to the grids. The grids were then manually backside blotted for 2–3 s (Whatman, Nr.41, UK) and plunge frozen by the Cryoplunge3 (Gatan, USA). Plunge freezing was done at RT with a humidity of roughly 80%. Frozen grids were examined and screened using a JEM2200FS microscope (JEOL, Japan).

### Data collection

The tomographic data were acquired on a 300 kV Titan Krios (Thermo Fisher, USA) at the ETH Zürich with a K2 camera and GIF‐Quantum energy filter (Gatan). Tilt series from −60° to 60° were collected with a 2° increment using a bidirectional tilt scheme starting from 0° for the data with ATP and unidirectional for the nucleotide‐free dataset using SerialEM (Mastronarde, 2005). The total electron dose used for both datasets was 60 electrons per Å^2^. The frames of the dose fractionated, normalized micrographs were aligned using the IMOD alignframes command. Tomograms were reconstructed using IMOD (Kremer *et al*, 1996; an example tomogram is shown in Appendix Fig S3A and cross section S3B). CTF correction was done in IMOD.

### Initial data processing—axoneme_aln

Subtomogram averaging was performed as described elsewhere (Bui & Ishikawa, 2013). This procedure includes manually picking of the MTDs in IMOD. The points along one MTD were interpolated and segmented into 24‐nm arrays. These subvolumes were aligned along one MTDs, assuming they have similar Euler angles. The 24‐nm subtomograms of all nine MTDs were then aligned based on the ninefold pseudo‐symmetry of the axoneme. For obtaining the 96‐nm subunit, the right frame was selected from the 24‐nm segments, to reduce redundancy. More details can be found in Bui and Ishikawa (Bui & Ishikawa, 2013).

### Refined data processing—RELION

To eliminate reference bias, reference‐free classification and refinement was applied in RELION (RELION version 2.1; Scheres, 2012). All the subtomograms were extracted with 24‐nm periodicity from the tomograms according to the aligned orientation and position determined by axoneme_aln, using BSOFT (Heymann, 2001). These prealigned subtomograms were imported into RELION, and autorefine was used to refine the alignment of the axoneme_aln output. Then, reference‐free 3D classification, without alignment, focused on the IDA and radial spokes and separately on one ODA of the 96‐nm repeat was carried out in RELION (Classification workflow in Appendix Fig S3C). A cubic mask generated with BSOFT included the three dynein heads and part of the tail complex for the ODA and another mask including all IDA and the radial spokes to separate the four different 96‐nm frames were applied. The outputs of the ODA classification were iteratively refined and reclassified with RELION. Since none of these classes represented the post‐PS conformation (apo), we used the data from a nucleotide‐free dataset and processed it in the same way as the data with ATP. The class with the mixture of intermediate structures was further classified using reference‐based classification focusing on the individual HC (Appendix Fig S3C, “reference based classification”). As references, the subtomogram averages generated in the previous iteration of the pre‐PS, post‐PS, and unmixed intermediate were used. The information of the 96‐nm frame and the ODA class was combined to generate a 96‐nm subtomogram average for the different classes (Appendix Fig S3C, “96‐nm average”). The FSC was calculated in RELION using the same mask as was used for the last refinement step. The resolution based on the FSC is 30 Å for the post‐PS conformation and 38 Å for the pre, 38 Å and 40 Å for the intermediate conformations, respectively (Appendix Fig S3D).

### Data interpretation and model building

The subaverages from the RELION jobs were visualized using IMOD, UCSF Chimera, and ChimeraX (Goddard *et al*, 2005; Pettersen *et al*, 2021). The recently published models of the ODA structure by single‐particle cryo‐EM (Walton *et al*, 2021; PDB‐7KZM; Kubo *et al*, 2021; PDB‐7MOQ; Rao *et al*, 2021; PDB‐7K58 and PDB‐7K5B) were fitted into our 24‐nm subtomogram average and 96‐nm subtomogram average maps maximizing real‐space cross‐correlation in UCSF Chimera. The models PBD‐7MOQ, PDB‐7KZM, and PDB‐7K58 were fitted as rigid bodies. The fit of the PDB‐7KZM dynein heads was improved by individual rigid body fitting of the dynein heads. For PDB‐7K5B, Coot (Emsley *et al*, 2010) was used for rigid body fitting of parts of the heavychains separately from the rest of the structure. Then, real‐space refinement with 5 Å self‐restraints and 6 Å local distance, secondary structure and all geometric restraints (torsion, planar, and trans peptide, and Ramachandran) was used to reconnect the broken chains from the step before. An overall refinement weight of 0.1 was used. For the pre‐PS structures, the crystallographic model of cytoplasmic dynein (PDB‐4RH7) (Schmidt *et al*, 2015) and the HC of the Shulin–ODA complex (PDB‐6ZYW) were fitted into the dynein head densities. With rigid body fitting and real‐space refinement, the best‐fitting pre‐PS dynein heads were used together with the PDB‐7MOQ tail complex to build a pseudo‐atomic model. The same restraints as for the post‐power stroke were applied. To validate the refinement, the atoms outside of envelope were analyzed. For visualization, the function Segger in Chimera was used to hide B‐tubule.

To analyze the differences between two adjacent MTDs in the different subclasses, the distances and angles between two adjacent subtomograms were calculated as shown in Appendix Fig S3E. For this, only particles with an adjacent partner were used. Unclassified particles lead to exclusion of the preceding subtomogram.

## Author contributions

**Noemi Zimmermann:** Conceptualization; resources; data curation; software; formal analysis; validation; investigation; visualization; methodology; writing – original draft; writing – review and editing. **Akira Noga:** Resources; data curation; writing – review and editing. **Jagan Mohan Obbineni:** Resources; software; supervision; funding acquisition; validation; investigation; visualization. **Takashi Ishikawa:** Conceptualization; data curation; software; formal analysis; supervision; funding acquisition; validation; investigation; visualization; methodology; project administration; writing – review and editing.

## Disclosure and competing interests statement

The authors declare that they have no conflict of interest.

## Supporting information



Appendix

Movie EV1

Movie EV2

Movie EV3

Movie EV4

## Data Availability

Coordinates of pseudo‐atomic models and cryo‐ET maps have been deposited in the Protein Data Bank under accession codes PDB‐8BX8 (https://www.rcsb.org/structure/8BX8) and the Electron Microscopy Data Bank (EMDB) EMD‐16312 (https://www.ebi.ac.uk/emdb/EMD‐16312) for the post‐PS, PDB‐8BWY (https://www.rcsb.org/structure/8BWY) and EMD‐16304 (https://www.ebi.ac.uk/emdb/EMD‐16304) for the pre‐PS state. The cryo‐ET maps of the intermediate state have been deposited at the Electron Microscopy Data Bank (EMDB) with the accession codes EMD‐16309 (https://www.ebi.ac.uk/emdb/EMD‐16309), EMD‐16310 (https://www.ebi.ac.uk/emdb/EMD‐16310).
