# Supplementary material for: ATP‐induced conformational change of axonemal outer dynein arms revealed by cryo‐electron tomography
Source: EMBO J. 2023 Apr 13;42(12):e112466. doi: 10.15252/embj.2022112466 (PMC10267688; doi:10.15252/embj.2022112466)
Supplement: Supplementary file 1 — Appendix [file EMBJ-42-e112466-s001.pdf]

Appendix Figures and Tables

For the manuscript

ATP induced conformational change of axonemal outer dynein arms revealed by cryo-electron tomography
Condensed title: ATP induced conformational change of ODA

Author:

Noemi Zimmermann[1], Akira Noga[1], Jagan Mohan Obbineni[1,2], Takashi Ishikawa[1]

[1] Paul Scherrer Institut (PSI), Laboratory of Nanoscale Biology, CH-5232 Villigen PSI, Switzerland [2]VIT School for Agricultural Innovations and Advanced, Learning (VAIAL), VIT, Vellore, India

Table of Contents

Appendix Materials

Contents	page
**Appendix Figure S1** Different types of dyneins	2
**Appendix Figure S2** Intra and inter ODA connection of the post-PS state.	3
**Appendix Figure S3** Data processing of subtomogram averaging and classification.	4
**Appendix Table S1** PDB data used for fitting and model building in this work.	5
**Appendix Table S2** Cryo-EM data collection statistics.	6
**Appendix Table S3** CC values between PDB structures and cryo-ET maps.	7

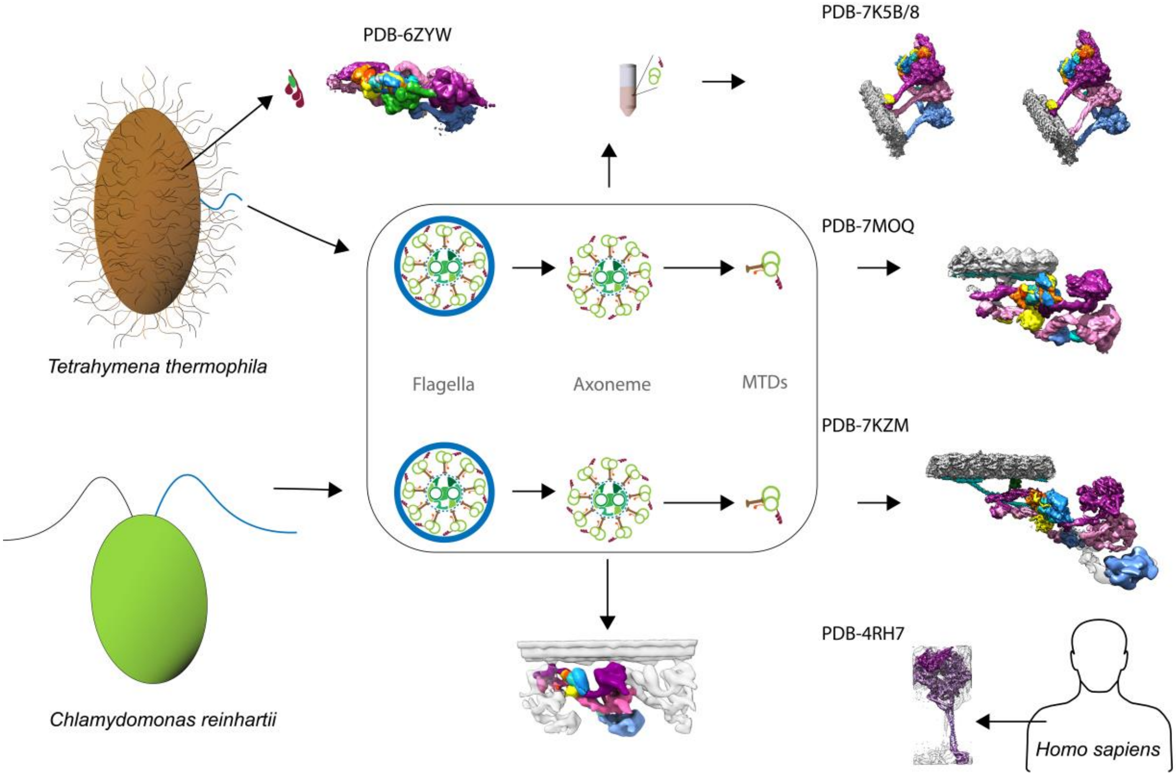

Appendix Figure S1 Different types of dyneins solved by crystallography (PDB-4RH7), cryo-EM (PDB-6ZYW, 7K5B/8, 7MOQ, 7KZM) and cryo-ET of Tetrahymena and Chlamydomonas. Axonemal dynein were solved packed by Shulin (PDB-6ZYW) and assembled on the A-tubule (PDB-7MOQ, 7KZM) or the B-tubule (PDB-7K5B/8).

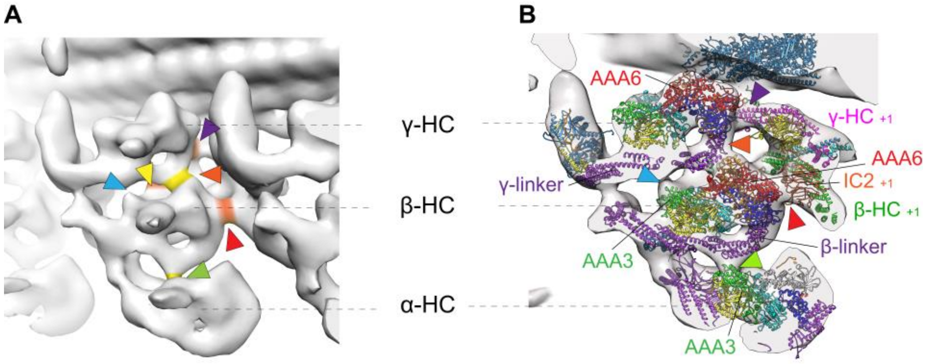

Appendix Figure S2 Intra and inter ODA connection of the post-PS state.

A) The intra- (orange) and inter-ODA (yellow) connections are highlighted.

B) Real space refined post-PS model fitted into the same view of A to indicate which proteins are involved in the inter- and intra-ODA connections. The following interactions are marked by arrow heads: purple-γ AAA6 to proximal γ tail, orange and light blue – γ linker to β head, yellow-β head to proximal NND, red-β head to proximal IC2, green – β linker to α head.

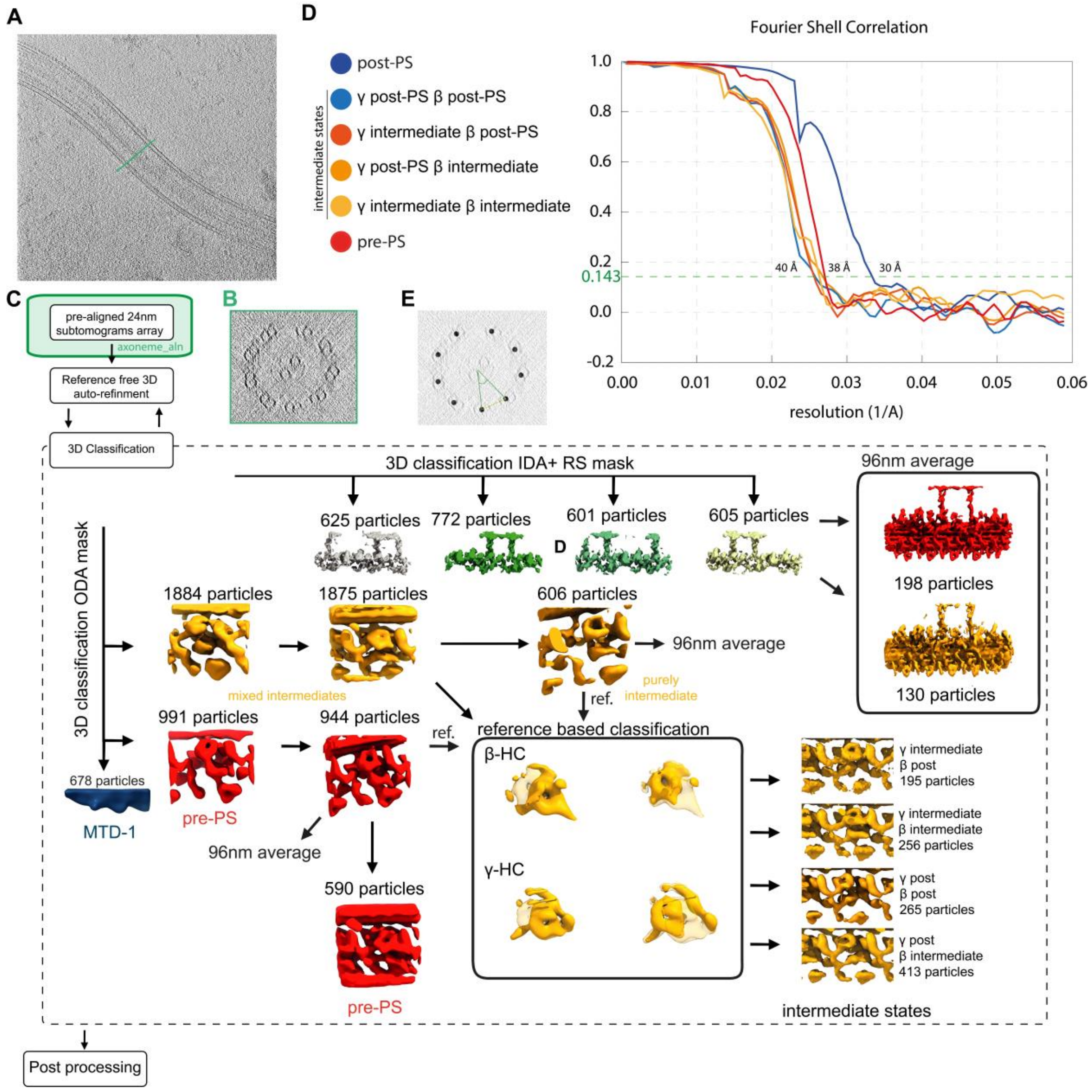

Appendix Figure S3 Data processing of subtomogram averaging and classification.

A-B) Slice though a tomogram of an axoneme (A) and a cross-section (B) indicated in green.

C) Pipeline of cryo-ET data processing. Two main software, axoneme_aln and RELION were used. The workflow of RELION 3D classification with pre-aligned 24nm subtomograms as an input shows that the data processing was split into two parts, IDA and radial spoke classification and ODA classification. Recombination of these two information lead to 96nm subtomogram average. The intermediate state with 1875 particles was further classified by a reference based classification, where a purely intermediate, post-PS and pre-PS conformation served as references.

D) Post-processing of the 24nm subtomogram average lead to a FSC of 30Å for post-PS and 38Å for pre-PS and 38-40 Å for the four intermediate states.

E) Schematics on how the distance and angle between two neighboring subtomograms were calculated.

Appendix Table S1. PDB data used for fitting and model building in this work.

PDB ID	reference	Image of the model/strucutre	*Specimen*	Nucleotide condition	Method of structure determination
7KZM	Walton et al.	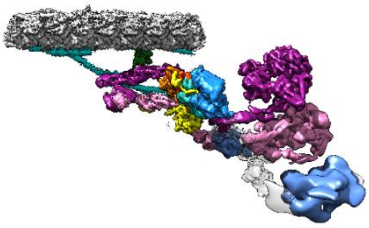	*Chlamydomonas* ODA anchored on A-tubule	No nucleotide	SPA
7MOQ	Kubo et al.	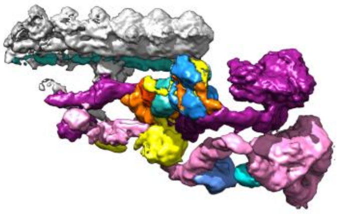	*Tetrahymena* ODA anchored on A-tubule	No nucleotide	SPA
7K5b	Rao et al.	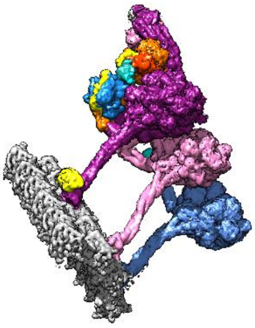	*Tetrahymena* ODA isolated and bound on B-tubule	No nucleotide	SPA
7K58	Rao et al.	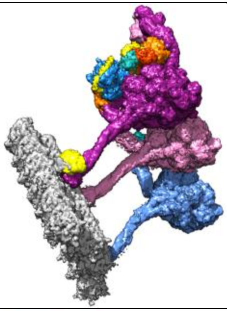	*Tetrahymena* ODA isolated and bound on B-tubule	No nucleotide	SPA
6ZYW	Mali et al.	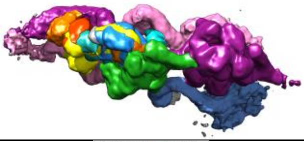	*Tetrahymena* ODA during IFT	ATP	SPA
4RH7	Schmidt et al.	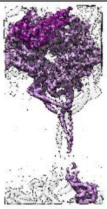	*Homo sapiens* pre-power stoke dynein	ATP-VO_4	crystallography

Appendix Table S2. Cryo-EM data collection statistics.

	WT (apo)	WT ATP	
		Pre subpopulation	Intermediate subpopulation
Data collection and processing			
Microscope	Titan Krios	Titan Krios	
Camera	Gatan K2 Summit	Gatan K2 Summit	
Magnification	53000x	33000x	
Voltage (kV)	300	300	
Number of frames per images	21	5	
Total electron exposure ($e^-/Å^2$)	80	80	
Defocus range	3-4	4-5	
Pixel size binned	8.49	8.5	
Box size	74	74	
Initial particle images	3167	3553	
Tomograms used	8	12	
Final particle images	2131	590	195,256,265,413
Map resolution (0.143 FS threshold)	30 Å	38 Å	39 Å, 40 Å, 38 Å,40 Å
EMDB	EMD-16312	EMD-16304	EMD-16310, EMD-16309

Appendix Table S3. CC values between PDB structures and cryo-ET maps.

Post-PS conformations			
	Original PDB model	Protofilaments removed	Modified PDB model
7KZM	0.8079	0.7454	0.7747*/0.8047**
7MOQ	0.8505	0.8	-
7K58	0.7701	0.6562	-
7K5B	0.6797	0.6797	0.8422 (MTBS3)
Pre-PS conformations			
	Original post-PS conformation		Modified pre-PS conformation
Post tail complex	0.8382	Pre tail complex	0.8443

** γ dynein head shift* *** γ and αdynein head shift*
