# Supplementary material for: ATP‐induced conformational change of axonemal outer dynein arms revealed by cryo‐electron tomography
Source: EMBO J. 2023 Apr 13;42(12):e112466. doi: 10.15252/embj.2022112466 (PMC10267688; doi:10.15252/embj.2022112466)
Supplement: Supplementary file 6 [file EMBJ-42-e112466-s003.pdf]

THE EMBO JOURNAL

ATP induced conformational change of axonemal outer dynein arms revealed by cryo-electron tomography

Noemi Zimmermann, Akira Noga, Jagan Obbineni, and Takashi Ishikawa
DOI: 10.15252/embj.2022112466

Corresponding author: Takashi Ishikawa (takashi.ishikawa@psi.ch)

Review Timeline:

Manuscript Transferred:	25th Aug 22
Editorial Decision:	29th Aug 22
Revision Received:	13th Sep 22
Editorial Decision:	10th Feb 23
Revision Received:	8th Mar 23
Accepted:	16th Mar 23

Editor: Ieva Gailite

Transaction Report:

Review COMMONS

This manuscript was transferred to The EMBO Journal following peer review at Review Commons.

(Note: With the exception of the correction of typographical or spelling errors that could be a source of ambiguity, letters and reports are not edited. Depending on transfer agreements, referee reports obtained elsewhere may or may not be included in this compilation. Referee reports are anonymous unless the Referee chooses to sign their reports.)

Review #1

1. Evidence, reproducibility and clarity:

Evidence, reproducibility and clarity (Required)

Summary

Zimmermann et al. provide a comparison between recent atomic models of the ODA determined by single particle cryo-EM and their conformation within intact axonemes by cryo-ET subtomogram averaging. They observed slight changes in the position of the motors for the structures of Kubo et al. and Walton et al., but the structure of Rao et al. required more changes, indicating that within the axoneme, the conformation of the ODA is influenced by the MTD on which it is docked, and the neighboring MTD to which its motors bind. They then use the information from their newly fit models to interpret cryo-ET maps of axonemes in the presence of ATP, which activates the ODA and other axonemal dyneins. They observe two states of the ODA, and describe how the position of the motor, linker, MTBD and LC tower change during the powerstroke cycle. A revised model of the ODA and the ability to describe conformational changes at the subunit level provides an advance on previous work and will be of interest to the dynein and cilia fields. However, the comments below must be addressed prior to publication, and additional work is needed to make the paper accessible.

Major comments

1. Greater clarity is needed in the introduction to explain the differences between the recent atomic models of the ODA. This is essential to understanding the paper, including Fig. 3. Arguably, the top half of Fig. S2 provides a stronger case for the study than any of the current main figures.

2. In the manuscript, potential differences between Chlamydomonas and Tetrahymena ODAs are not considered but need to be explored. Comparison of Tetrahymena models within Chlamydomonas maps could result in misinterpretations.

3. Systematic quantification of the fit-to-map should be provided for the models before and after refitting (together with evidence - see the point below - that the model has not been inappropriately distorted to fit the map). This information could be inserted into an expanded Supplementary Table.

4. Because the revised pseudo-atomic model of the ODA is a chimera of PDBs from different organisms, it does not accurately represent the Chlamydomonas ODA. The modeling method also has the potential to introduce clashes between rigid-body fitted chains. Validation of the model is necessary, and alternative approaches to generate a more accurate model (e.g. AlphaFold and molecular dynamics flexible fitting) should be considered.

5. Additional evidence needs to be provided to demonstrate that the intermediate state observed

in Figure 4 is robustly detected and does not simply represent the data that doesn't fall into the "good" classes. In Fig. S1, the map looks very noisy and requires denoising. Are there other changes observed in the IDAs that would support the existence of an intermediate state?

6. The speculation that the additional density bound to a-HC is Lis1 is not well-supported. Lis1 binds AAA4/5 (PDB: 5VH9), not AAA2/3. The fit of the Lis1 homolog into the cryo-ET density does not appear consistent with Lis1 binding the motor. The authors should consider other possibilities that could explain the additional density.

Minor comments

1. The results section "Post-PS structure and Fitting of the atomic models" is very dense. It should be split into subsections to help guide the reader through specific models or regions of the ODA.

2. ODA numbering should be made consistent with previous papers (i.e. ODA1-4 as in Bui et al., 2012)

3. The ODA-shulin model (PDB: 6ZYW) is inaccurately described as the state transported during IFT, but experimental confirmation of this hypothesis is lacking.

4. The term TTH for tail-to-head contacts is too similar to T/TH for the tether/tetherhead complex and should be changed. An abbreviation may not be necessary.

5. Please check to make sure that all figures and figure legends clearly specify which map/model/motor is being shown. This will make the figures easier to follow.

6. The structures in Fig. 3 are from Rao et al., not Walton et al.

7. Fig 5M-O is very difficult to interpret. Could the authors consider coloring by region, for one of the maps, or at least put the maps in a similar orientation to the ODA cores as in Fig 2?

8. The final processing step in panel Fig S1B is confusing. Additional information is needed to explain the supervised classification step and how the final particle set was derived.

9. Atomic resolution should not be used to describe structures determined to 4.3 Å resolution (e.g. EMD-11579).

10. Supervised classification is not a method of validation

11. Please check for grammatical and spelling errors throughout the manuscript.

2. Significance:

Significance (Required)

While previous literature has interpreted ODA conformation in broad regions, this study goes farther by using recent atomic models to identify specific subunits that change conformations and interactions during the powerstroke. From my perspective as a structural biologist in the cilia field, I think this paper provides a conceptual advance to the study and interpretation of axonemes.

3. How much time do you estimate the authors will need to complete the suggested revisions:

Estimated time to Complete Revisions (Required)

(Decision Recommendation)

Between 1 and 3 months

4. Review Commons values the work of reviewers and encourages them to get credit for their work. Select 'Yes' below to register your reviewing activity at Publons; note that the content of your review will not be visible on Publons.

Reviewer Publons

No

Review #2

1. Evidence, reproducibility and clarity:

Evidence, reproducibility and clarity (Required)

- The authors report cryo-EM tomography of the axoneme of motile cilia in the presence and absence of ATP, providing new insight into the mechanism of action of the motors. They use crystal structures and information from single particle cryo-EM to fit these fragments into their new density obtained in situ, and show that distortions to these smaller structures are required for them to be accommodated in the complex and crowded environment of the axoneme. The movies provided show the relevant fits in 3D, which is important because the complexity of the structures makes 2D visualisations limited. Are the authors sufficiently confident in their atomistic models that they would be useful for other researchers, and if so are they planning to release them (e.g. as pdb files) with the paper, or on request?

- There are potentially a few editorial additions and changes that the authors might consider making to improve the readability of the paper for non-specialists in the axoneme. For example, could they insert a sentence explaining what Shulin is and its biological significance?
There are numerous abbreviations and acronyms throughout the manuscript - would it be helpful to maybe write some of those out in full where appropriate?
In the very helpful Supplementary table containing the pdb IDs used to fit into the current structure, would it be useful to have a small picture of each system as one of the columns in this table?
Would it also potentially be helpful to include a figure summarising the different types of dynein observed in this and other relevant studies - e.g the pre and post-powerstroke states, Shulin bound etc? This would help the reader to understand the magnitudes of the conformational changes between these various states that are under discussion.
Could a schematic diagram representing the "winch" and "rotation" models be included potentially? In the Discussion section, I was not able to understand whether the winch or rotation models are most supported by the data in this paper, or whether a mixture of the two might be needed to understand axoneme mechanics, so further clarification of this would be helpful.

- Please note that all of these comments are suggestions to improve accessibility and readability, and are not essential additions for the paper to be publishable.

CROSS-CONSULTATION COMMENTS

I was very interested to read the detailed and informative comments from the other referees. While I agree with referee 1 point 4 that the use of alpha-fold to predict how atomistic structures from different organisms may differ, and subsequent flexible fitting would be desirable, this in my opinion would be an enormous amount of work, and would be best reserved for subsequent publications. Sharing of the pdb files of the fitted structures obtained so far would open this mammoth task up to the rest of the community.

Given the complexity of the axoneme, and the huge amount of expertise needed to obtain and process these tomograms, I did wonder if this community would consider forming a collaborative consortium where researchers worked together to construct a common model.

2. Significance:

Significance (Required)

- The paper reports more complete and detailed structural information on the axoneme than (to my knowledge) has been obtained before. The fitting of atomistic level structures into the density to create a pseudo-atomic model is highly instructive.

- To me, it was not in the least bit surprising that distortions from the structures obtained in isolation using single particle analysis are required for an optimal fit. In fact, theoretical work reported by Richardson et al, QRB 2020 showed for inner dynein arms that the crowded environment provided solely by the microtubule tracks within the axoneme modified the

conformations of the dynein stalk that were accessible compared to a simple isolated dynein motor. While this study considers outer dynein arms, the conceptual physical rationale is equivalent to the findings here.
In my opinion, the finding reported in this paper that considering fragments of biological ultrastructures is not necessarily equivalent to the whole functioning entity is both important and profound, and has implications beyond motile cilia, particularly as cryo-electron tomography enables us to visualise ever larger and more complex functional biological assemblies.

- Please note that my area of expertise does not enable me to comment on the experimental procedures used to obtain the tomograms, as I am a computer modeller with an interest in dynein.

3. How much time do you estimate the authors will need to complete the suggested revisions:

Estimated time to Complete Revisions (Required)

(Decision Recommendation)

Between 1 and 3 months

4. Review Commons values the work of reviewers and encourages them to get credit for their work. Select 'Yes' below to register your reviewing activity at Publons; note that the content of your review will not be visible on Publons.

Reviewer Publons

Yes

Review #3

1. Evidence, reproducibility and clarity:

Evidence, reproducibility and clarity (Required)

Summary:

The study attempts to reconcile cryo-EM SPA structures of ODAs with in situ tomographic

reconstructions.

Several key discrepancies between SPA structures and the native in situ structures (here) are highlighted in the study with a particular focus on the positions of various ODA motor head components (linker, tails etc.) during the powerstroke cycle.

The study also highlights largely concordant inter and intra-ODA connections between previous SPA structures and the tomographic reconstructions.

Major comments:

Overall, the key conclusions are convincing. No additional experiments are suggested. The manuscript is acceptable provided minor comments below are addressed.

Minor comments:

The text could be improved throughout for improved clarity. Overall, the figures are good, but some panels are over-annotated which is confusing. Simplification or cartoon illustrations could add clarity to the figures.

CROSS-CONSULTATION COMMENTS

The paper still represents a significant and sufficient advance. Correction of factual errors flagged up by other reviewers (use of correct references and citations, correct species for Lis1 models used etc.) is required and essential prior to acceptance. Addition of more details in the sample preparation methods section would also be useful. Depositing PDBs and maps is recommended.

Agree on the overall point of improving accessibility and readability of the text. Figures can be much improved to highlight the biological insights for the reader.

The point of contention between extra density corresponding to either Lis1 or LC5 is valid. Tempering the assertion and removing bias towards Lis1 in the text would resolve this issue. The authors are putting forth a speculative model which is valid; this model can be tested in future work.

Several minor comments highlighted by other reviewers are fair and should be addressed as best as possible.

Several major comments highlighted by other reviewers (specifically: use of structure prediction and modeling, filament distortion analysis etc.) are well beyond the scope of the present work and do not advance the specific and main conclusions of the current study.

2. Significance:

Significance (Required)

The study presents structures of ODAs during their powerstroke cycles in situ in their native context and integrates previous structural models of ODAs to provide novel insights.

The identification of a Lis1 or LC5 like density adjacent to the alpha-HC and observation of a curved position of the beta-HC stalk in the native state adds further novelty to the study.

The study will be of interest to researchers like myself working on cilia motility and dynein motors.

3. How much time do you estimate the authors will need to complete the suggested revisions:

Estimated time to Complete Revisions (Required)

(Decision Recommendation)

Less than 1 month

4. Review Commons values the work of reviewers and encourages them to get credit for their work. Select 'Yes' below to register your reviewing activity at Publons; note that the content of your review will not be visible on Publons.

Reviewer Publons

Yes

Review #4

1. Evidence, reproducibility and clarity:

Evidence, reproducibility and clarity (Required)

In the manuscript "ATP induced conformational change of axonemal outer dynein arms studied by cryo-electron tomography", Noemi Zimmermann et al. built pseudo atomic models of outer

dynein arms (ODA) from the native axonemes with and without ATP treatment using a combination of cryo-electron tomography (cryo-ET), sub-tomogram averaging and atomic model fitting analysis. The authors clearly distinguished several important conformations of ODA using their high-quality cryo-ET maps. The authors showed that in situ ODA conformation in post-power stroke state is different from in vitro ODA structures, either lacking B-tubule binding or A-tubule binding. In my opinion, this is a very important observation by taking the advantage of cryo-ET analysis on intact axonemes. Furthermore, by freezing the activated axoneme immediately after ATP treatment, the authors obtained the active pre-power stroke and an intriguing intermediate conformation of ODA. By generating pseudo atomic models, the authors were able to compare the structural changes in dynein heads and stalks among different states and highlighted geometrical constraints from neighboring MTDs on ODA. Overall, the findings by Noemi et al have provided really exciting insights into ODA conformational changes during the power stoke. I therefore highly recommend publication of the manuscript. However, before the official publication, I do have some comments and believe the authors can further improve their manuscript to make it more exciting to the field.

1. The authors only showed the maps from sub-tomogram averages (Supply Fig 1). I suggest the authors also show a representative reconstruction of the whole tomogram as a supplementary figure so that we have a better overview of the reconstruction.
2. Since this is a typical piece of structural work, I highly suggest the authors summarize their cryo-ET data collection and processing parameters as a supplementary table, such as standard microscopy parameters, image pixel sizes, number of tomograms, number of particles etc.
3. On page 5 and Supplementary Figure 2H, I, the authors fitted Lis1 model to the additional density at the interface between AAA2 and AAA3. This is really intriguing. However, according the currently published Lis1-dynein structures (PMID: 28886386, PMID: 34994688), it seems that Lis1 interacts with dynein on AAA4 and AAA5. Can the authors discuss anything about the evolutionary conservation of Lis1 binding? In addition, the authors did not fit LC5 model into the density map. I am a bit worried that there might be some bias on Lis1. With the fast development of protein prediction tools like Alphafold and Rosetta fold, the authors would be able to have a nice prediction of the LC5 structure to fit the additional density. I therefore suggest the authors try to do so if it is technically feasible, and then discuss a bit more on this point.
4. On Page 6, the authors mentioned that "neither of the two structures (MTBS1, MTBS2) represented our conformation of ODA". This is an interesting finding since in the reconstituted ODA array on MTD by Rao et al., 2021 paper, they observed both MTBS1 (γMTBD: 0 nm; βMTBD:0nm; αMTBD:8nm) and MTBS2 (γMTBD: 0 nm; βMTBD:8nm; αMTBD:8 nm) conformations (Here, 0nm and 8nm represent the relative longitudinal positions along the tubulin lattice among the three MTBDs). According to the post-ODA structure from this manuscript, the authors found all three heavy chains are in the post-2 states, or equivalently with MTBDs at the 8-nm position (γMTBD: 8nm; βMTBD:8nm; αMTBD:8nm, Fig3G). The authors also mentioned that the conformations of minimum energy of ODA are different in vivo and in vitro in the discussion. On the other hand, many structures previously determined by X-ray and EM in vitro show that Post-1 were overwhelmingly preferred before Rao et al reported the Post-2 state. This raises a very interesting question, how many MTBS states can ODA actually adopt in vivo? In theory, the three MTBDs can be arranged in at least a certain subset of the eight states

(000,001,010,100,011,101,110,111) if the distance between any two MTBDs is restricted to 8nm, and the movement of each MTBD is restricted along one direction. There might be more states if the movement is more than one step. Therefore, from the results of both this manuscript and Rao et al., 2021 paper, probably not all states could have been observed. I wonder if the authors can perform more 3D classification on their STA particles in the post-PS state to demonstrate and see if there is any chance to see more states in vivo. I was a bit surprised because I felt there might be more states in vivo than in vitro reconstitution. The idea that the two neighboring MTDs can restrict the ODA conformation is great. I suggest the authors discuss more about the possible effects from two neighboring instead of just a general concept of energy minimization (probably it is impossible to estimate the total energy of such a complex system under physiological conditions using any kind of currently available techniques).

5. In Figure 4, the authors observe structural changes of ODA among different states. The figures clearly show the differences among post-PS, intermediate state, and pre-PS state. For the pre-PS and intermediate state, I wonder if the authors can map the two conformations back onto the raw tomograms and show how they look like in a relatively large region with more repeating units.

6. In Figure 4, I really appreciate the authors pointing out the distortion (changes in distances and the rotation angles) between adjacent MTDs. To my knowledge, the distortion of neighboring MTDs during ODA power stroke cycle has not been well analyzed in many previous publications. To gain more insights on this part, I wonder if the authors can perform more quantitative analysis on all adjacent MTDs with and without ATP from their current data sets. There are some nice publications on filament distortion analysis using single particle approaches, including one from the Sindelar lab (PMID: 32636254, Fig 4 and 6). More specifically, since the authors already have the position and Euler angle information of each particle from the subtomogram averaging, it is possible to extract the distortion information from two adjacent MTDs. After extracting distortion information from all MTD pairs and plotting the data points in different ways, the authors may be able to correlate the ODA conformation, MTD bending and see whether they could find some intriguing patterns. The authors do not have to incorporate all their results from this analysis into the current manuscript since there are already many interesting things, but briefly showing some curvature distribution would be highly appreciated, and the authors can still publish other interesting results in their future publications.

7. It seems the authors have not deposited their maps and PDBs (as they are XXXX's in the current manuscript). It would be nice to if they can do so at their earliest convenience.

8. On page 5, the authors found an additional density next to the α dynein which could be Lis1 or LC5 (see also minor comment #1). Again, this is an advantage using cryo-ET. This observation is also missing from ODA SPA papers, and I appreciate the authors for the careful examination. Since there are several 96-nm MTD maps from previously studies from Chlamydomonas and Tetrahymena, I wonder if this additional density is also present from previous cryo-ET maps.

9. On page 5, the sentence "one unit of the dimeric Homo sapiens Lis1 (PDB-5VLJ (Htet et al., 2020, p. 1)) and fitting it into our density allowed us to assess its likeability." The Lis1 model in PDB-5VLJ is from Saccharomyces cerevisiae, not from Home sapiens. In addition, the reference paper doesn't match the PDB-5VLJ. The authors should cite the correct paper.

10. On page 6 Figure 2 legend D, B HC should be β HC.

11. On page 8 Figure 3 legend "A and B) Rigid body fit of the whole MTBS1 map (Walton et al., 2021).". The citation here should be Rao et al., 2021.

12. In Figure 5, the authors generated models for the pre-PS conformation of ODA. From the cryo-ET density map, the authors suggested that β-MTBD was in a bent conformation, which

was similar to the conformation in shulin-ODA. This is a novel observation. Since the authors have atomic models, I suggest the authors directly use the PDB models for better visualization of structural changes among post-PS ODA, intermediate ODA, and pre-PS ODA. A supplementary figure or movie will be very nice.
13. On page 16 "EM grids" session, I suggest the authors provide slightly more details on their sample preparation, such as the concentration of the axoneme, blotting time, temperature, humidity etc.

2. Significance:

Significance (Required)

Significance: This is a very nice manuscript for better understanding of the motile cilia system. It is a significant progress in the field with lots of interesting findings.

Audience: People in the field of dynein, motile cilia, cytoskeleton and in cryo-ET technique as well.

My expertise: I am very confident in reviewing this paper, both biologically and technically, and I have recently published in this field as well.

3. How much time do you estimate the authors will need to complete the suggested revisions:

Estimated time to Complete Revisions (Required)

(Decision Recommendation)

Between 1 and 3 months

4. Review Commons values the work of reviewers and encourages them to get credit for their work. Select 'Yes' below to register your reviewing activity at [Publons](); note that the content of your review will not be visible on Publons.

Reviewer Publons

No

Revision Plan

Manuscript number: RC-xx-xx
Corresponding author(s): First name, Last name

[The "revision plan" should delineate the revisions that authors intend to carry out in response to the points raised by the referees. It also provides the authors with the opportunity to explain their view of the paper and of the referee reports.

The document is important for the editors of affiliate journals when they make a first decision on the transferred manuscript. It will also be useful to readers of the reprint and help them to obtain a balanced view of the paper.

If you wish to submit a full revision, please use our "Full Revision" template. ***It is important to use the appropriate template to clearly inform the editors of your intentions.****]*

1. General Statements [optional]

We appreciate positive and constructive evaluation, points, proposal and criticisms from the reviewers. We are now working on revision process. Many points are already addressed in the attached preliminary revision, while some revisions are on-going. We present our revision and plan of further revision below.

2. Description of the planned revisions

3. Description of the revisions that have already been incorporated in the transferred manuscript

Here we describe our response to all the points from the four reviewers in the order. The points from the reviewers are in black and our responses are in red.

Reviewer #1
1. Greater clarity is needed in the introduction to explain the differences between the recent atomic models of the ODA. This is essential to understanding the paper, including Fig. 3.
We will rewrite the text and captions to identify each atomic model clearly.
Arguably, the top half of Fig. S2 provides a stronger case for the study than any of the current main figures.
We integrated the top half of Fig. S2 into Fig.2 to present our post-PS model in the as visible as Reviewer #1 requested.
More importantly we replaced our pseudo-atomic model of ODA in Fig.2A-E with another model of us. The model shown in the new Fig.2A-E is the same as Fig.3, which was obtained by

remodeling Rao et al. (instead of Kubo et al. and Walton et al.) to avoid chimera modeling from Chlamydomonas and Tetrahymena.

2. In the manuscript, potential differences between Chlamydomonas and Tetrahymena ODAs are not considered but need to be explored. Comparison of Tetrahymena models within Chlamydomonas maps could result in misinterpretations.
We will discuss in detail about difference of dynein structures between the two species in Discussion.

3. Systematic quantification of the fit-to-map should be provided for the models before and after refitting (together with evidence - see the point below - that the model has not been inappropriately distorted to fit the map). This information could be inserted into an expanded Supplementary Table.
We added Supplementary Table 3 to show cross correlation between atomic models (Bui, Brown, Zhang groups and our modeling) and our cryo-ET density map, which demonstrates improvement of fitting by our modeling.
4. Because the revised pseudo-atomic model of the ODA is a chimera of PDBs from different organisms, it does not accurately represent the Chlamydomonas ODA. The modeling method also has the potential to introduce clashes between rigid-body fitted chains. Validation of the model is necessary, and alternative approaches to generate a more accurate model (e.g. AlphaFold and molecular dynamics flexible fitting) should be considered.
Fitting of individual chains did indeed lead to clashes between the chains. We therefore used the whole ODA model and use Coot with restrains to rigid body fit sub chains of the same model and did local real space refinement. We will update our model in PDB-Dev.

5. Additional evidence needs to be provided to demonstrate that the intermediate state observed in Figure 4 is robustly detected and does not simply represent the data that doesn't fall into the "good" classes.
We will provide another figure to prove that the intermediate structure is not a mixture of failure subclasses. In case a subaverage is a mixture of multiple classes, it appears with a notable structure (such as ring structure of dynein) as two structures overlapped or blurred. We will present a map of dynein ring in the intermediate conformation clearly
In Fig. S1, the map looks very noisy and requires denoising. Are there other changes observed in the IDAs that would support the existence of an intermediate state?
We will replace Fig. S1 with denoised surface rendering. IDA analysis required 4 times more datasets and thus in the scope of our next work.

6. The speculation that the additional density bound to a-HC is Lis1 is not well-supported. Lis1 binds AAA4/5 (PDB: 5VH9), not AAA2/3. The fit of the Lis1 homolog into the cryo-ET density does not appear consistent with Lis1 binding the motor. The authors should consider other

possibilities that could explain the additional density.
We agree with Reviewer #1 that there is not enough evidence to interpret this density as Lis1 and appreciate this reviewer. We are now working computationally to find possible protein components for this density. We modeled Chlamydomonas Lis1 and another candidate LC5 using Alphafold2 and are conducting docking simulation to assess how likely these proteins are to be in this location.

Minor comments 1. The results section "Post-PS structure and Fitting of the atomic models" is very dense. It should be split into subsections to help guide the reader through specific models or regions of the ODA.
We will rewrite this section.

2. ODA numbering should be made consistent with previous papers (i.e. ODA1-4 as in Bui et al., 2012)
We will revise ODA numbering throughout the text.

3. The ODA-shulin model (PDB: 6ZYW) is inaccurately described as the state transported during IFT, but experimental confirmation of this hypothesis is lacking.

We rewrote the two sentences "Recently it was found that the ODA are transported to the axoneme …. This structure was then solved to atomic resolution (Mali et al., 2021)" to avoid confusion. The new description is

A recent work revealed that in *Tetrahymena* during reciliation of the ODA, the ODA is in a closed conformation, where the DHY3 (γ HC analog) is folded onto the other two HC (Mali et al., 2021). This conformation is held together and inhibited by Shulin.
Related topics in Introduction were also rewritten.

4. The term TTH for tail-to-head contacts is too similar to T/TH for the tether/tetherhead complex and should be changed. An abbreviation may not be necessary.
We fixed the text to mention tail-to-head contact without abbreviations.

5. Please check to make sure that all figures and figure legends clearly specify which map/model/motor is being shown. This will make the figures easier to follow.
We will doublecheck in this respect.

6. The structures in Fig. 3 are from Rao et al., not Walton et al.
We fixed this mistake.

7. Fig 5M-O is very difficult to interpret. Could the authors consider coloring by region, for one of the maps, or at least put the maps in a similar orientation to the ODA cores as in Fig 2?

We are now remaking the figure for more clarity.

8. The final processing step in panel Fig S1B is confusing. Additional information is needed to explain the supervised classification step and how the final particle set was derived.

We will update Fig. S1B for clarification of classification procedure.

9. Atomic resolution should not be used to describe structures determined to 4.3 Å resolution (e.g. EMD-11579).

We will make sure that the different maps and models are referred to in the right way.

10. Supervised classification is not a method of validation

We removed “validation” from the first sentence of the section “Supervised classification” in Methods and described more precisely how we used supervised classification in this analysis

11. Please check for grammatical and spelling errors throughout the manuscript.

We will ask a native speaker for proof-reading.

Reviewer #2

Are the authors sufficiently confident in their atomistic models that they would be useful for other researchers, and if so are they planning to release them (e.g. as pdb files) with the paper, or on request?

We will upload our model in PDB-Dev.

There are potentially a few editorial additions and changes that the authors might consider making to improve the readability of the paper for non-specialists in the axoneme. For example, could they insert a sentence explaining what Shulin is and its biological significance?

It is linked to the minor comment #3 of Reviewer 1. We rewrote the sentences to introduce shulin as an important protein to bind ODA for priming transportation in cilia.

There are numerous abbreviations and acronyms throughout the manuscript - would it be helpful to maybe write some of those out in full where appropriate?

It is linked to the minor comment #4 of Reviewer 1. We spelled out these abbreviations.

In the very helpful Supplementary table containing the pdb IDs used to fit into the current structure, would it be useful to have a small picture of each system as one of the columns in this table?

We will update the table accordingly.

Would it also potentially be helpful to include a figure summarising the different types of dynein observed in this and other relevant studies - e.g the pre and post-powerstroke states, Shulin bound etc? This would help the reader to understand the magnitudes of the conformational changes between these various states that are under discussion.

We will try to make such an introductory figure.

Could a schematic diagram representing the "winch" and "rotation" models be included potentially?
We would cite Fig.5 from Ishikawa (2015) Cilia 4:3 for graphical representation of winch and rotation hypotheses. We will describe carefully in Discussion, since the new knowledge of dynein conformational change, in which the stalk changes its direction not only along the PF, but also perpendicularly, will not allow a simple “winch or rotation” argument.
In the Discussion section, I was not able to understand whether the winch or rotation models are most supported by the data in this paper, or whether a mixture of the two might be needed to understand axoneme mechanics, so further clarification of this would be helpful.
The change of the angle between the stalk and the head observed in this work shows more preference to the winch model – the stalk extends toward the proximal direction in both pre- and post-PS structures. However, it is not anymore a simple in-plane movement parallel to MTD. We will describe clearly in Results and Discussion in the full-revision.
I was very interested to read the detailed and informative comments from the other referees. While I agree with referee 1 point 4 that the use of alpha-fold to predict how atomistic structures from different organisms may differ, and subsequent flexible fitting would be desirable, this in my opinion would be an enormous amount of work, and would be best reserved for subsequent publications.
We agree with this reviewer. We think the flexible fitting will be possible with higher spatial resolution of our analysis in the future. In this work, our modeling was done with each component as a rigid body, with exception of dynein heavy chains, in which we allowed flexibility between subdomains, such as the stalk, the tail and the ring (but not within each subdomain).
Sharing of the pdb files of the fitted structures obtained so far would open this mammoth task up to the rest of the community.
We are depositing our model in PDB-Dev upon submission of the full revision.

Reviewer #3
The text could be improved throughout for improved clarity.
We will revise the whole text to find unclear sections and try to clarify them. Additionally we will ask a native speaker for proof-reading.

Overall, the figures are good, but some panels are over-annotated which is confusing. Simplification or cartoon illustrations could add clarity to the figures.
We are in the process to fix panels with over-annotation.
Addition of more details in the sample preparation methods section would also be useful.
We revised the sample preparation section and added details of light microscopy and blotting conditions

Depositing PDBs and maps is recommended.
We are depositing our model in PDB-Dev and subtomogram averaging map in EMDB.

Revision Plan

Reviewer #4

1. The authors only showed the maps from sub-tomogram averages (Supply Fig 1). I suggest the authors also show a representative reconstruction of the whole tomogram as a supplementary figure so that we have a better overview of the reconstruction.

We will add a supplementary figure to show the whole tomogram.

2. Since this is a typical piece of structural work, I highly suggest the authors summarize their cryo-ET data collection and processing parameters as a supplementary table, such as standard microscopy parameters, image pixel sizes, number of tomograms, number of particles etc.

We added Supplementary table 2 for cryo-EM parameters.

3. On page 5 and Supplementary Figure 2H, I, the authors fitted Lis1 model to the additional density at the interface between AAA2 and AAA3. This is really intriguing. However, according the currently published Lis1-dynein structures (PMID: 28886386, PMID: 34994688), it seems that Lis1 interacts with dynein on AAA4 and AAA5. Can the authors discuss anything about the evolutionary conservation of Lis1 binding? In addition, the authors did not fit LC5 model into the density map. I am a bit worried that there might be some bias on Lis1. With the fast development of protein prediction tools like Alphafold and Rosetta fold, the authors would be able to have a nice prediction of the LC5 structure to fit the additional density. I therefore suggest the authors try to do so if it is technically feasible, and then discuss a bit more on this point.

This comment is linked to comment 6 of Reviewer #1 and we appreciate both reviewers. To be fair to discuss possible protein identification, we are now conducting molecular docking simulation to assess likeliness of binding of Lis1 and LC5. We did molecular modeling of Chlamydomonas Lis1 and LC5 by Alphafold2. Now we are docking them to dynein heavy chains. We hope we can add the result in the full revision and discuss our interpretation.

4. On Page 6, the authors mentioned that "neither of the two structures (MTBS1, MTBS2) represented our conformation of ODA". This is an interesting finding since in the reconstituted ODA array on MTD by Rao et al., 2021 paper, they observed both MTBS1 (γMTBD: 0 nm; βMTBD:0nm; αMTBD:8nm) and MTBS2 (γMTBD: 0 nm; βMTBD:8nm; αMTBD:8 nm) conformations (Here, 0nm and 8nm represent the relative longitudinal positions along the tubulin lattice among the three MTBDs). According to the post-ODA structure from this manuscript, the authors found all three heavy chains are in the post-2 states, or equivalently with MTBDs at the 8-nm position (γMTBD: 8nm; βMTBD:8nm; αMTBD:8nm, Fig3G). The authors also mentioned that the conformations of minimum energy of ODA are different in vivo and in vitro in the discussion. On the other hand, many structures previously determined by X-ray and EM in vitro show that Post-1 were overwhelmingly preferred before Rao et al reported the Post-2 state. This raises a very interesting question, how many MTBS states can ODA actually adopt in vivo? In theory, the three MTBDs can be arranged in at least a certain subset of the eight states (000,001,010,100,011,101,110,111) if the distance between any two MTBDs is restricted to 8nm, and the movement of each MTBD is restricted along one direction. There

might be more states if the movement is more than one step. Therefore, from the results of both this manuscript and Rao et al., 2021 paper, probably not all states could have been observed. I wonder if the authors can perform more 3D classification on their STA particles in the post-PS state to demonstrate and see if there is any chance to see more states in vivo. I was a bit surprised because I felt there might be more states in vivo than in vitro reconstitution. The idea that the two neighboring MTDs can restrict the ODA conformation is great. I suggest the authors discuss more about the possible effects from two neighboring instead of just a general concept of energy minimization (probably it is impossible to estimate the total energy of such a complex system under physiological conditions using any kind of currently available techniques).
We could not find subclasses by further classification. They ended up with classification based on missing wedge directions. Since Post-2 structure dominates our in-situ structures, we think there is a factor actively biasing the equilibrium. We will discuss about this mechanism further in the full revision.

5. In Figure 4, the authors observe structural changes of ODA among different states. The figures clearly show the differences among post-PS, intermediate state, and pre-PS state. For the pre-PS and intermediate state, I wonder if the authors can map the two conformations back onto the raw tomograms and show how they look like in a relatively large region with more repeating units.
We will provide raw tomograms with ODA states mapped, in the full revision.

6. In Figure 4, I really appreciate the authors pointing out the distortion (changes in distances and the rotation angles) between adjacent MTDs. To my knowledge, the distortion of neighboring MTDs during ODA power stroke cycle has not been well analyzed in many previous publications. To gain more insights on this part, I wonder if the authors can perform more quantitative analysis on all adjacent MTDs with and without ATP from their current data sets. There are some nice publications on filament distortion analysis using single particle approaches, including one from the Sindelar lab (PMID: 32636254, Fig 4 and 6). More specifically, since the authors already have the position and Euler angle information of each particle from the subtomogram averaging, it is possible to extract the distortion information from two adjacent MTDs.
We are now carrying out a quantitative analysis of axonemal geometry at different nucleotide conditions. We could prove the geometry of nine MTDs are more disturbed in the presence of ATP. We will provide this data in the full revision.
After extracting distortion information from all MTD pairs and plotting the data points in different ways, the authors may be able to correlate the ODA conformation, MTD bending and see whether they could find some intriguing patterns. The authors do not have to incorporate all their results from this analysis into the current manuscript since there are already many interesting things, but briefly showing some curvature distribution would be highly appreciated, and the authors can still publish other interesting results in their future publications.
As reviewer #4 mentions, we would like to involve further analysis of distribution of ODA conformations with correlation to ciliary curvature in the next work, since we need more data to

demonstrate significant correlation and freezing environment, which retains physiological beating of cilia (since in any cryo-ET work until now, axonemes are frozen after blotting by filter paper, physiological beating during freezing is not confirmed).

7. It seems the authors have not deposited their maps and PDBs (as they are XXXX's in the current manuscript). It would be nice to if they can do so at their earliest convenience.
We will deposit our map to PDB-Dev at the time of full submission.

8. On page 5, the authors found an additional density next to the α dynein which could be Lis1 or LC5 (see also minor comment #1). Again, this is an advantage using cryo-ET. This observation is also missing from ODA SPA papers, and I appreciate the authors for the careful examination. Since there are several 96-nm MTD maps from previously studies from Chlamydomonas and Tetrahymena, I wonder if this additional density is also present from previous cryo-ET maps.
We will make a research on previous 3D works and comment in the revision.

9. On page 5, the sentence "one unit of the dimeric Homo sapiens Lis1 (PDB-5VLJ (Htet et al., 2020, p. 1)) and fitting it into our density allowed us to assess its likeability." The Lis1 model in PDB-5VLJ is from Saccharomyces cerevisiae, not from Home sapiens. In addition, the reference paper doesn't match the PDB-5VLJ. The authors should cite the correct paper.
Thank you for pointing out. We will fix the citation.

10. On page 6 Figure 2 legend D, B HC should be β HC.
We fixed

11. On page 8 Figure 3 legend "A and B) Rigid body fit of the whole MTBS1 map (Walton et al., 2021).". The citation here should be Rao et al., 2021.
We fixed this mistake.

12. In Figure 5, the authors generated models for the pre-PS conformation of ODA. From the cryo-ET density map, the authors suggested that β-MTBD was in a bent conformation, which was similar to the conformation in shulin-ODA. This is a novel observation. Since the authors have atomic models, I suggest the authors directly use the PDB models for better visualization of structural changes among post-PS ODA, intermediate ODA, and pre-PS ODA. A supplementary figure or movie will be very nice.
We will make such a supplementary figure.

13. On page 16 "EM grids" session, I suggest the authors provide slightly more details on their sample preparation, such as the concentration of the axoneme, blotting time, temperature, humidity etc.
We revised the cryo-preparation part of Methods.

Revision Plan

4. Description of analyses that authors prefer not to carry out

N/A. We think we can address all the points from the four reviewers.

1st Editorial Decision 29th Aug 2022

Thank you for submitting your manuscript for consideration by the EMBO Journal. I have now read your manuscript, the reviewer comments and your response to them. Based on our editorial assessment and the referees' positive evaluations, I would like to invite you to submit a revised version of the manuscript along the lines indicated in your revision plan.

We generally allow three months as standard revision time. As a matter of policy, competing manuscripts published during this period will not negatively impact on our assessment of the conceptual advance presented by your study. However, please contact me as soon as possible upon publication of any related work to discuss the appropriate course of action. Should you foresee a problem in meeting this three-month deadline, please let us know in advance in order to arrange an extension.

When preparing your letter of response to the referees' comments, please bear in mind that this will form part of the Review Process File and will therefore be available online to the community. For more details on our Transparent Editorial Process, please visit our website: https://www.embopress.org/page/journal/14602075/authorguide#transparentprocess. Please also see the attached instructions for further guidelines on preparation of the revised manuscript.

Please feel free to contact me if you have any further questions regarding the revision. Thank you for the opportunity to consider your work for publication. I look forward to receiving your revised manuscript.

Point-by-point response to the reviewers

ATP-induced conformational change of axonemal outer dynein arms studied by cryo-electron tomography

Authors:

Noemi Zimmermann[1], Akira Noga[1], Jagan Mohan Obbineni[1,2], Takashi Ishikawa[1]

Reviewer #1

1. Greater clarity is needed in the introduction to explain the differences between the recent atomic models of the ODA. This is essential to understanding the paper, including Fig. 3.

We newly made Supplementary figure 1 and supplementary table 1 to compare the conformation and preparation conditions of all the 3D structures we fitted in this work. We also rewrote Introduction and captions to identify each atomic model clearly.

Arguably, the top half of Fig. S2 provides a stronger case for the study than any of the current main figures.

We integrated the top half of Fig. S2 into Fig.2 to present our post-PS model as visible as Reviewer #1 requested.

2. In the manuscript, potential differences between Chlamydomonas and Tetrahymena ODAs are not considered but need to be explored. Comparison of Tetrahymena models within Chlamydomonas maps could result in misinterpretations.

We thank this reviewer to point out this issue. One major difference between heavy chains from these species is located between the linker and the neck parts (~500-~900 in sequence). It is missing in the genome of Tetrahymena (~350 amino acids). However, we do not see significant structural difference between our structure and Tetrahymena ODA from our group and from Nicastro's lab (EMD-7805 from doi:10.1091/mbc.E18-06-0405). We discussed this point of dynein structures between the two species in Discussion (end of the paragraph "The minimum energy …" in Discussion.

3. Systematic quantification of the fit-to-map should be provided for the models before and after

refitting (together with evidence - see the point below - that the model has not been inappropriately distorted to fit the map). This information could be inserted into an expanded Supplementary Table.

We added Supplementary Table 3 to show cross correlation between atomic models (Bui, Brown, Zhang groups and our modeling) and our cryo-ET density map, which demonstrates improvement of fitting by our modeling.

4. Because the revised pseudo-atomic model of the ODA is a chimera of PDBs from different organisms, it does not accurately represent the Chlamydomonas ODA. The modeling method also has the potential to introduce clashes between rigid-body fitted chains. Validation of the model is necessary, and alternative approaches to generate a more accurate model (e.g. AlphaFold and molecular dynamics flexible fitting) should be considered.

Fitting of individual chains did indeed lead to clashes between the chains. We therefore used the whole ODA model and use Coot with restrains to rigid body fit sub chains of the same model and did local real space refinement. We deposited our updated model in PDB. We also attempted docking by Haddock to reduce clashes, but this attempt did not bring significant improvements.

5. Additional evidence needs to be provided to demonstrate that the intermediate state observed in Figure 4 is robustly detected and does not simply represent the data that doesn't fall into the "good" classes.

We thank the reviewers to point out. We made further classification of subtomograms, which were once assigned as the intermediate-state structure and found that there are multiple structures involved. We interpreted them as multiple intermediate structures and provide them as another figure (new Fig.7). They consist of various combinations of three heavy chains – all three in intermediate conformation, two in intermediate and one in post etc.

In Fig. S1, the map looks very noisy and requires denoising. Are there other changes observed in the IDAs that would support the existence of an intermediate state?

We replace the noisy panels in Fig. S1 with denoised surface rendering. IDA analysis required 4 times more datasets and thus in the scope of our next work.

6. The speculation that the additional density bound to a-HC is Lis1 is not well-supported. Lis1 binds AAA4/5 (PDB: 5VH9), not AAA2/3. The fit of the Lis1 homolog into the cryo-ET density does not appear consistent with Lis1 binding the motor. The authors should consider other possibilities that could explain the additional density.
We agree with Reviewer #1 that there is not enough evidence to interpret this density as Lis1 and appreciate this reviewer. In the revised manuscript, we mention possibility to assign this density to Lis1 and another candidate LC5 equally, only as possible candidates.

Minor comments 1. The results section "Post-PS structure and Fitting of the atomic models" is very dense. It should be split into subsections to help guide the reader through specific models or regions of the ODA.

We rewrote this part and separated into several subsections with subtitles.

2. ODA numbering should be made consistent with previous papers (i.e. ODA1-4 as in Bui et al., 2012)

We revised ODA numbering throughout the text.

3. The ODA-shulin model (PDB: 6ZYW) is inaccurately described as the state transported during IFT, but experimental confirmation of this hypothesis is lacking.

We rewrote the two sentences "Recently it was found that the ODA are transported to the axoneme …. This structure was then solved to atomic resolution (Mali et al., 2021)" to avoid confusion. The new description is

A recent work revealed that in *Tetrahymena* during reciliation of the ODA, the ODA is in a closed conformation, where the DHY3 (γ HC analog) is folded onto the other two HC (Mali et al., 2021). This conformation is held together and inhibited by Shulin.

Related topics in Introduction were also rewritten.

A recent work revealed that in *Tetrahymena* during intraflagellar transport (IFT) of the ODA, the ODA is in a closed conformation, where the DHY3 (γ HC analog) is folded onto the other two HC (Mali et al., 2021). → A recent work revealed that in *Tetrahymena* during reciliation the ODA is in a closed conformation, where the DHY3 (γ HC analog) is folded onto the other two HC (Mali et al., 2021).

4. The term TTH for tail-to-head contacts is too similar to T/TH for the tether/tetherhead complex and should be changed. An abbreviation may not be necessary.

We fixed the text to mention tail-to-head contact without abbreviations.

5. Please check to make sure that all figures and figure legends clearly specify which map/model/motor is being shown. This will make the figures easier to follow.

We fixed the text, referring figures, and legends in this respect.

6. The structures in Fig. 3 are from Rao et al., not Walton et al.

We fixed this mistake.

7. Fig 5M-O is very difficult to interpret. Could the authors consider coloring by region, for one of the maps, or at least put the maps in a similar orientation to the ODA cores as in Fig 2?

We remade the figure for more clarity. In the new figure, pre- and post-structures from the modeled atomic structure are colored differently and juxtaposed over our pre- and post-cryo-ET maps.

8. The final processing step in panel Fig S1B is confusing. Additional information is needed to explain the supervised classification step and how the final particle set was derived.

We updated this panel (now Fig. S3B) for clarification of reference based classification procedure.

9. Atomic resolution should not be used to describe structures determined to 4.3 Å resolution (e.g. EMD-11579).

In the revised version, we do not use the word “atomic” for the 4.3A resolution structure.

10. Supervised classification is not a method of validation

We removed the paragraph “supervised classification”, which includes the word “validation” (we agree with the reviewer) in Methods.

11. Please check for grammatical and spelling errors throughout the manuscript.

Native speakers made a proof-reading.

Reviewer #2

Are the authors sufficiently confident in their atomistic models that they would be useful for other researchers, and if so are they planning to release them (e.g. as pdb files) with the paper, or on request?

We uploaded our models and maps in PDB and EM-Databank (mentioned in the end of the text).

There are potentially a few editorial additions and changes that the authors might consider making to improve the readability of the paper for non-specialists in the axoneme. For example, could they insert a sentence explaining what Shulin is and its biological significance?

It is linked to the minor comment #3 of Reviewer 1. We rewrote the sentences to introduce shulin as an important protein to bind ODA for priming transportation in cilia.

There are numerous abbreviations and acronyms throughout the manuscript - would it be helpful to maybe write some of those out in full where appropriate?

It is linked to the minor comment #4 of Reviewer 1. We spelled out these abbreviations.

In the very helpful Supplementary table containing the pdb IDs used to fit into the current structure, would it be useful to have a small picture of each system as one of the columns in this table?

We added supplementary table 1 for this purpose accordingly.

Would it also potentially be helpful to include a figure summarising the different types of dynein observed in this and other relevant studies - e.g the pre and post-powerstroke states, Shulin bound etc? This would help the reader to understand the magnitudes of the conformational changes between these various states that are under discussion.

We added supplementary figure 1.

Could a schematic diagram representing the "winch" and "rotation" models be included potentially?

In the new version, we cited Fig.1 from Ueno et al. (2008) PNAS which describes Winch and Rotation models in the best way by graphical representation. We described carefully in Discussion, since the new knowledge of dynein conformational change, in which the stalk changes its direction not only along the PF, but also perpendicularly, will not allow a simple “winch or rotation” argument.

In the Discussion section, I was not able to understand whether the winch or rotation models are most supported by the data in this paper, or whether a mixture of the two might be needed to understand axoneme mechanics, so further clarification of this would be helpful.
The change of the angle between the stalk and the head observed in this work shows more preference to the winch model – the stalk extends toward the proximal direction in both pre- and post-PS structures. However, it is not anymore a simple in-plane movement parallel to MTD. We described in this way in Discussion (in the paragraph “In all the three states…”) in the revised version.

I was very interested to read the detailed and informative comments from the other referees. While I agree with referee 1 point 4 that the use of alpha-fold to predict how atomistic structures from different organisms may differ, and subsequent flexible fitting would be desirable, this in my opinion would be an enormous amount of work, and would be best reserved for subsequent publications.

We agree with this reviewer. We attempted model refinement by Haddock, but it did not improve our modeling. We think the flexible fitting will be possible with higher spatial resolution of our analysis in the future. In this work, our modeling was done with each component as a rigid body, with exception of dynein heavy chains, in which we allowed flexibility between subdomains, such as the stalk, the tail and the ring (but not within each subdomain).

Sharing of the pdb files of the fitted structures obtained so far would open this mammoth task up to the rest of the community.
We deposited our models in PDB and maps in EM-databank.

Reviewer #3

The text could be improved throughout for improved clarity.

We revised the whole text to fix unclear point and try to clarify them. Additionally we asked native speakers for proof-reading.

Overall, the figures are good, but some panels are over-annotated which is confusing. Simplification or cartoon illustrations could add clarity to the figures.
We fixed over-annotated panels such as Fig.6CD and Fig.7IJ or moved them to Supplementary figures (Fig.2KL to Fig.S2).

Addition of more details in the sample preparation methods section would also be useful.

We revised the sample preparation section and added details of light microscopy and blotting conditions

Depositing PDBs and maps is recommended.
We deposited our models in PDB and subtomogram averaging maps in EMDB.

Reviewer #4

1. The authors only showed the maps from sub-tomogram averages (Supply Fig 1). I suggest the authors also show a representative reconstruction of the whole tomogram as a supplementary figure so that we have a better overview of the reconstruction.

We added supplementary figure 3AB to show the whole tomogram.

2. Since this is a typical piece of structural work, I highly suggest the authors summarize their cryo-ET data collection and processing parameters as a supplementary table, such as standard microscopy parameters, image pixel sizes, number of tomograms, number of particles etc.

We added Supplementary table 3 for cryo-EM parameters.

3. On page 5 and Supplementary Figure 2H, I, the authors fitted Lis1 model to the additional density at the interface between AAA2 and AAA3. This is really intriguing. However, according the currently published Lis1-dynein structures (PMID: 28886386, PMID: 34994688), it seems that Lis1 interacts with dynein on AAA4 and AAA5. Can the authors discuss anything about the evolutionary conservation of Lis1 binding? In addition, the authors did not fit LC5 model into the density map. I am a bit worried that there might be some bias on Lis1. With the fast development of protein prediction tools like Alphafold and Rosetta fold, the authors would be able to have a nice prediction of the LC5 structure to fit the additional density. I therefore suggest the authors try to do so if it is technically feasible, and then discuss a bit more on this point.

This comment is linked to comment 6 of Reviewer #1 and we appreciate both reviewers. To be fair to discuss possible protein identification, we conducted molecular docking by Haddock to assess likeliness of binding of Lis1 and LC5. We did molecular modeling of Chlamydomonas Lis1 and LC5 by Alphafold2. However we could not obtain results positively supporting one protein (such as Lis1) to be fitted better than other. We rewrote this part to be fairly describing possible candidate proteins for this unassigned density.

4. On Page 6, the authors mentioned that "neither of the two structures (MTBS1, MTBS2) represented our conformation of ODA". This is an interesting finding since in the reconstituted ODA array on MTD by Rao et al., 2021 paper, they observed both MTBS1 (γMTBD: 0 nm; βMTBD:0nm; αMTBD:8nm) and MTBS2 (γMTBD: 0 nm; βMTBD:8nm; αMTBD:8 nm) conformations (Here, 0nm and 8nm represent the relative longitudinal positions along the tubulin lattice among the three MTBDs). According to the post-ODA structure from this manuscript, the authors found all three heavy chains are in the post-2 states, or equivalently with MTBDs at the 8-nm position (γMTBD: 8nm; βMTBD:8nm; αMTBD:8nm, Fig3G). The authors also mentioned that the conformations of minimum energy of ODA are different in vivo and in vitro in the discussion. On the other hand, many structures previously determined by X-ray and EM in vitro show that Post-1 were overwhelmingly preferred before Rao et al reported the Post-2 state. This raises a very interesting question, how many MTBS states can ODA actually adopt in vivo? In theory, the three MTBDs can be arranged in at least a certain subset of the eight states (000,001,010,100,011,101,110,111) if the distance between any two MTBDs is restricted to 8nm, and the movement of each MTBD is restricted along one direction. There might be more states if the movement is more than one step. Therefore, from the results of both this manuscript and Rao et al., 2021 paper, probably not all states could have been observed. I wonder if the authors can perform more 3D classification on their STA particles in the post-PS state to demonstrate and see if there is any chance to see more states in vivo. I was a bit surprised because I felt there might be more states in vivo than in vitro reconstitution. The idea that the two neighboring MTDs can restrict the ODA conformation is great. I suggest the authors discuss more about the possible effects from two neighboring instead of just a general concept of energy minimization (probably it is impossible to estimate the total energy of such a complex system under physiological conditions using any kind of currently available techniques).

We could not find subclasses by further classification. They ended up with classification based on missing wedge directions. Since Post-2 structure dominates our in-situ structures, we think there is a factor actively biasing the equilibrium. We discussed about this mechanism in Discussion (the paragraph beginning with “The minimum evergy conformation…”).

5. In Figure 4, the authors observe structural changes of ODA among different states. The figures clearly show the differences among post-PS, intermediate state, and pre-PS state. For the pre-PS and intermediate state, I wonder if the authors can map the two conformations back onto the raw tomograms and show how they look like in a relatively large region with more repeating units.

We mapped distribution of the conformations of ODA on tomograms. There are axonemes which show interesting patterns of distribution, while others show almost random distribution. We have

not gotten enough number of tomograms to further interpret our current results. We decided not to show them in the current manuscript and leave this topic in the next work.

6. In Figure 4, I really appreciate the authors pointing out the distortion (changes in distances and the rotation angles) between adjacent MTDs. To my knowledge, the distortion of neighboring MTDs during ODA power stroke cycle has not been well analyzed in many previous publications. To gain more insights on this part, I wonder if the authors can perform more quantitative analysis on all adjacent MTDs with and without ATP from their current data sets. There are some nice publications on filament distortion analysis using single particle approaches, including one from the Sindelar lab (PMID: 32636254, Fig 4 and 6). More specifically, since the authors already have the position and Euler angle information of each particle from the subtomogram averaging, it is possible to extract the distortion information from two adjacent MTDs.

We described statistics of geometries of adjacent MTDs in Results (paragraph beginning with “Alignment of the different tomographic maps …”). We are planning to discuss relation between global geometry of cilia (such as curvature), geometry of nine MTD (distance and angles of doublets) and states of dynein conformational changes in the next work, but think it is too early now.

After extracting distortion information from all MTD pairs and plotting the data points in different ways, the authors may be able to correlate the ODA conformation, MTD bending and see whether they could find some intriguing patterns. The authors do not have to incorporate all their results from this analysis into the current manuscript since there are already many interesting things, but briefly showing some curvature distribution would be highly appreciated, and the authors can still publish other interesting results in their future publications.

As reviewer #4 mentions, we would like to involve further analysis of distribution of ODA conformations with correlation to ciliary curvature in the next work, since we need more data to demonstrate significant correlation and freezing environment, which retains physiological beating of cilia (since in any cryo-ET work until now, axonemes are frozen after blotting by filter paper, physiological beating during freezing is not confirmed).

7. It seems the authors have not deposited their maps and PDBs (as they are XXXX's in the current manuscript). It would be nice to if they can do so at their earliest convenience.

We deposited our coordinates and maps to PDB and EM Databank.

8. On page 5, the authors found an additional density next to the α dynein which could be Lis1 or LC5 (see also minor comment #1). Again, this is an advantage using cryo-ET. This observation is also missing from ODA SPA papers, and I appreciate the authors for the careful examination. Since there are several 96-nm MTD maps from previously studies from Chlamydomonas and Tetrahymena, I wonder if this additional density is also present from previous cryo-ET maps.

This unassigned density is found in Tetrahymena cryo-ET (Nicastro’s group EMD-7805 from Zhao et al. 2018 Mol Biol Cell 29, 2566) as well. It is either common binding protein or a part of dynein HC, which is fluctuating and thus cannot be seen in the SPA map.

9. On page 5, the sentence "one unit of the dimeric Homo sapiens Lis1 (PDB-5VLJ (Htet et al., 2020, p. 1)) and fitting it into our density allowed us to assess its likeability." The Lis1 model in PDB-5VLJ is from Saccharomyces cerevisiae, not from Home sapiens. In addition, the reference paper doesn't match the PDB-5VLJ. The authors should cite the correct paper.

Thank you for pointing out. We modeled Chlamydomonas Lis1 by Alphafold2 and tried to fit this density. However, we could not obtain quantitative output to support this assignment. We decided to remove this sentence.

10. On page 6 Figure 2 legend D, B HC should be β HC.

We fixed.

11. On page 8 Figure 3 legend "A and B) Rigid body fit of the whole MTBS1 map (Walton et al., 2021).". The citation here should be Rao et al., 2021.

We fixed this mistake.

12. In Figure 5, the authors generated models for the pre-PS conformation of ODA. From the cryo-ET density map, the authors suggested that β-MTBD was in a bent conformation, which was similar to the conformation in shulin-ODA. This is a novel observation. Since the authors have atomic models, I suggest the authors directly use the PDB models for better visualization of structural changes among post-PS ODA, intermediate ODA, and pre-PS ODA. A supplementary figure or movie will be very nice.

We made a supplementary figure 3 to highlight change of stalk orientations. Movie 5 also presents change between pre- and post-conformations. In this movie, intermediate conformation is not shown, since, due to lower resolution, we could fit dynein HC model to our intermediate map, but could not model the entire ODA, as we did for pre- and post-structures.

13. On page 16 "EM grids" session, I suggest the authors provide slightly more details on their sample preparation, such as the concentration of the axoneme, blotting time, temperature, humidity etc.
We revised the cryo-preparation part of Methods and described more detail.

From the editor

1. Please find attached a document with comments and questions from the data editors. Please address the issues raised, keeping the changes tracked, and upload it as the manuscript text.
We checked. We accepted all the editing by the editor.

2. Please add up to five keywords.

We did.

3. Please move the Data Availability section to the end of the Methods and add reviewer access.

We described it in the end of Methods.

4. We noticed that the funding project IDs in the manuscript and in our system don't match. Please double-check and update as appropriate.

We fixed it.

5. CRediT has replaced the traditional author contributions section because it offers a systematic machine readable author contributions format that allows for more effective research assessment. Please remove the Authors Contributions from the manuscript and use the free text boxes beneath each contributing author's name in our system to add specific details on the author's contribution. More information is available in our guide to authors.

We removed it.

6. Please update the heading of the corresponding section to Disclosure and competing interests statement.

We updated it.

7. Please correct the format of the references list to 10 author names before et al and remove the DOIs.

We fixed it.

8. Please add short legends in simple README or docx format to the movies and zip each legend with the corresponding movie file.

We did.

9. Please add a table of contents with page numbers to the file with the suppl. figures and legends. Please rename the file "Appendix" and correct the nomenclature to "Appendix Figure S1" etc. and "Appendix Table S1". Please also group the files by type (figures and tables).

We changed these names as instructed.

10. Please ensure that all main figure panels are called out and are in the correct sequence; we were unable to find callouts for Fig 1D ; Fig 2A-E,H,J,L-M,O,R-S ; Fig 3F ; Fig 4C ; Fig 5A ; Fig 7A-B ; Appendix Table S1 and S2. Suppl. Fig 3 is called out before Suppl. Fig 2, please correct the sequence.

We did. Now all the figure panels, supplementary figures and tables are called in the order.

11. Please correct the heading "Figure Captions" to " Figure Legends". Suppl. Figures S1-3 are uploaded as separate files and are also part of the appendix -- please remove the files that are uploaded separately.

Thank you for submitting a revised version of your manuscript. I sincerely apologise for the unusually protracted assessment process due to delays in referee report submission. Your study has now been seen by two of the original referees, who find that their previous concerns have been addressed and now recommend publication of the manuscript. There remain only a few editorial points that have to be addressed before I can extend formal acceptance of the manuscript:

1. Please address the minor points by the referees. Reviewer #3 has also provided a more detailed textual editing of the text that I have attached to this email.

Please let me know if you have any questions regarding any of these points. You can use the link below to upload the revised files.

Thank you again for giving us the chance to consider your manuscript for The EMBO Journal. I look forward to receiving the final version.

Referee #2:

The manuscript by Zimmermann et al is much improved. I would recommend publication with minor revisions.
1. Real space refinement settings (i.e. set resolution) should be specified and statistics be provided.

2. A few grammatical suggestions:

...whereas eight HCs make an array in the inner dynein arm (IDA).

... whereas an IDA has regulatory functions (Kamiya, 2002).

... two intermediate (IC1 and IC2) and multiple light chains (LC1-LC10, including LC7a/b).

... but contact the B-tubule 1-2 nm more distal than the tomographic map suggests (Figure 2A,B).

... no sign of stalks crossing, unlike the pre-PS (Appendix Figure 3)

Referee #3:

- general summary and opinion about the principle significance of the study, its questions and findings

Key differences between ODA structures obtained by cryo-EM SPA and in situ cryo-ET are highlighted. This is the only work which provides a comprehensive view of ODAs in their native context and is therefore an important piece of literature to advance existing knowledge in the field.

- specific major concerns essential to be addressed to support the conclusions

None

- minor concerns that should be addressed

The authors have largely taken reviewer comments on board and addressed them satisfactorily. For example, the addition of supplementary figure 1 and supplementary table 1 are very helpful and would serve as a good resource for the field.

However, there remain several typographical errors dotted throughout the text and in some figure labels. This reviewer has attempted to proofread the manuscript for the authors as much as possible and provided the suggested corrections as well as some comments to the editor who may pass them onto the authors.

Figure 8 (if present is only referred to once in the text in the last line of results section before discussion; figure 8L). I strongly suspect this is a mistake.

- any additional non-essential suggestions for improving the study (which will be at the author's/editor's discretion)

Supplementary Figure 3 provides an excellent summary of the different states of the ODA and could be moved in the main figure panels and incorporated into the final summary figure/model (figure 7 or 8?). In this reviewers opinion, this could greatly enhance the citability of this study.

This reviewer suggests the authors look into protein Q22MS1 from Tetrahymena to speculate identity of the novel alpha-HC associated density or aid its identification in future studies.

In conclusion, this reviewer recommends acceptance of the manuscript with suggested minor changes.

Referee #2:

1. Real space refinement settings (i.e. set resolution) should be specified and statistics be provided.

We specified some refinement parameters in Methods. To note, we could not use some parameters such as Ramachandran-plot for statistics, because we used them for constraint.

2. A few grammatical suggestions:

...whereas eight HCs make an array in the inner dynein arm (IDA).

... whereas an IDA has regulatory functions (Kamiya, 2002).

... two intermediate (IC1 and IC2) and multiple light chains (LC1-LC10, including LC7a/b).

... but contact the B-tubule 1-2 nm more distal than the tomographic map suggests (Figure 2A,B).

... no sign of stalks crossing, unlike the pre-PS (Appendix Figure 3)

We corrected all these points. We thank Reviewer #2 for pointing them out.

Referee #3:

However, there remain several typographical errors dotted throughout the text and in some figure labels. This reviewer has attempted to proofread the manuscript for the authors as much as possible and provided the suggested corrections as well as some comments to the editor who may pass them onto the authors.

We appreciated this reviewers support and help. We fixed the text following this reviewers advices.

Figure 8 (if present is only referred to once in the text in the last line of results section before discussion; figure 8L). I strongly suspect this is a mistake.
This was corrected. Now all the panels of Figure 8 are mentioned in the text.

Supplementary Figure 3 provides an excellent summary of the different states of the ODA and could be moved in the main figure panels and incorporated into the final summary figure/model (figure 7 or 8?). In this reviewers opinion, this could greatly enhance the citability of this study.

We totally agree with Reviewer #3's suggestion. We moved Fig.S3 to the main text. It is now Figure 8G.

This reviewer suggests the authors look into protein Q22MS1 from Tetrahymena to speculate identity of the novel alpha-HC associated density or aid its identification in future studies.

We appreciate Reviewer #3 for this advice.

Thank you for addressing the final editorial points. I am now pleased to inform you that your manuscript has been accepted for publication in the EMBO Journal.

EMBO Press Author Checklist

Corresponding Author Name: Takashi Ishikawa	
Journal Submitted to: EMBO Journal	
Manuscript Number: EMBOJ-2022-112466R	

USEFUL LINKS FOR COMPLETING THIS FORM
[The EMBO Journal - Author Guidelines]
[EMBO Reports - Author Guidelines]
[Molecular Systems Biology - Author Guidelines]
[EMBO Molecular Medicine - Author Guidelines]

Reporting Checklist for Life Science Articles (updated January 2022)
This checklist is adapted from Materials Design Analysis Reporting (MDAR) Checklist for Authors. MDAR establishes a minimum set of requirements in transparent reporting in the life sciences (see Statement of Task: 10.31222/osf.io/9sm4x). Please follow the journal's guidelines in preparing your manuscript.
Please note that a copy of this checklist will be published alongside your article.

Abridged guidelines for figures
1. Data
The data shown in figures should satisfy the following conditions:
- the data were obtained and processed according to the field's best practice and are presented to reflect the results of the experiments in an accurate and unbiased manner.
- ideally, figure panels should include only measurements that are directly comparable to each other and obtained with the same assay.
- plots include clearly labeled error bars for independent experiments and sample sizes. Unless justified, error bars should not be shown for technical replicates.
- if n<5, the individual data points from each experiment should be plotted. Any statistical test employed should be justified.
- Source Data should be included to report the data underlying figures according to the guidelines set out in the authorship guidelines on Data Presentation.

2. Captions
Each figure caption should contain the following information, for each panel where they are relevant:
- a specification of the experimental system investigated (eg cell line, species name).
- the assay(s) and method(s) used to carry out the reported observations and measurements.
- an explicit mention of the biological and chemical entity(ies) that are being measured.
- an explicit mention of the biological and chemical entity(ies) that are altered/varied/perturbed in a controlled manner.
- the exact sample size (n) for each experimental group/condition, given as a number, not a range;
- a description of the sample collection allowing the reader to understand whether the samples represent technical or biological replicates (including how many animals, litters, cultures, etc.).
- a statement of how many times the experiment shown was independently replicated in the laboratory.
- definitions of statistical methods and measures:
  - common tests, such as t-test (please specify whether paired vs. unpaired), simple χ2 tests, Wilcoxon and Mann-Whitney tests, can be unambiguously identified by name only, but more complex techniques should be described in the methods section;
  - are tests one-sided or two-sided?
  - are there adjustments for multiple comparisons?
  - exact statistical test results, e.g., P values = x but not P values < x;
  - definition of 'center values' as median or average;
  - definition of error bars as s.d. or s.e.m.

Please complete ALL of the questions below.
Select "Not Applicable" only when the requested information is not relevant for your study.

Materials

Newly Created Materials	Information included in the manuscript?	In which section is the information available? (Reagents and Tools Table, Materials and Methods, Figures, Data Availability Section)
New materials and reagents need to be available; do any restrictions apply?	Not Applicable	

Antibodies	Information included in the manuscript?	In which section is the information available? (Reagents and Tools Table, Materials and Methods, Figures, Data Availability Section)
For **antibodies** provide the following information:<br>- Commercial antibodies: RRID (if possible) or supplier name, catalogue number and or/clone number<br>- Non-commercial: RRID or citation	Not Applicable	

DNA and RNA sequences	Information included in the manuscript?	In which section is the information available? (Reagents and Tools Table, Materials and Methods, Figures, Data Availability Section)
**Short novel DNA or RNA including primers, probes**: provide the sequences.	Not Applicable	

Cell materials	Information included in the manuscript?	In which section is the information available? (Reagents and Tools Table, Materials and Methods, Figures, Data Availability Section)
**Cell lines:** Provide species information, strain. Provide accession number in repository **OR** supplier name, catalog number, clone number, and/**OR** RRID.	Yes	Materials and Methods
**Primary cultures:** Provide species, strain, sex of origin, genetic modification status.	Not Applicable	
Report if the cell lines were recently **authenticated** (e.g., by STR profiling) and tested for mycoplasma contamination.	Not Applicable	

Experimental animals	Information included in the manuscript?	In which section is the information available? (Reagents and Tools Table, Materials and Methods, Figures, Data Availability Section)
**Laboratory animals or Model organisms:** Provide species, strain, sex, age, genetic modification status. Provide accession number in repository **OR** supplier name, catalog number, clone number, **OR** RRID.	Not Applicable	
**Animal observed in or captured from the field:** Provide species, sex, and age where possible.	Not Applicable	
Please detail **housing and husbandry conditions**.	Not Applicable	

Plants and microbes	Information included in the manuscript?	In which section is the information available? (Reagents and Tools Table, Materials and Methods, Figures, Data Availability Section)
**Plants:** provide species and strain, ecotype and cultivar where relevant, unique accession number if available, and source (including location for collected wild specimens).	Not Applicable	
**Microbes:** provide species and strain, unique accession number if available, and source.	Not Applicable	

Human research participants	Information included in the manuscript?	In which section is the information available? (Reagents and Tools Table, Materials and Methods, Figures, Data Availability Section)
If collected and within the bounds of privacy constraints report on age, sex and gender or ethnicity for all study participants.	Not Applicable	

Core facilities	Information included in the manuscript?	In which section is the information available? (Reagents and Tools Table, Materials and Methods, Figures, Data Availability Section)
If your work benefited from core facilities, was their service mentioned in the acknowledgments section?	Yes	Acknowledgement

Design

Study protocol	Information included in the manuscript?	In which section is the information available? (Reagents and Tools Table, Materials and Methods, Figures, Data Availability Section)
If study protocol has been **pre-registered, provide DOI in the manuscript**. For clinical trials, provide the trial registration number **OR** cite DOI.	Not Applicable	
Report the **clinical trial registration number** (at ClinicalTrials.gov or equivalent), where applicable.	Not Applicable	

Laboratory protocol	Information included in the manuscript?	In which section is the information available? (Reagents and Tools Table, Materials and Methods, Figures, Data Availability Section)
Provide DOI OR other citation details if **external detailed step-by-step protocols** are available.	Yes	Materials and Methods

Experimental study design and statistics	Information included in the manuscript?	In which section is the information available? (Reagents and Tools Table, Materials and Methods, Figures, Data Availability Section)
Include a statement about **sample size** estimate even if no statistical methods were used.	Yes	Materials and Methods
Were any steps taken to minimize the effects of subjective bias when allocating animals/samples to treatment (e.g. **randomization procedure**)? If yes, have they been described?	Not Applicable	
Include a statement about **blinding** even if no blinding was done.	Not Applicable	
Describe **inclusion/exclusion criteria** if samples or animals were excluded from the analysis. Were the criteria pre-established? <br>If sample or data points were omitted from analysis, report if this was due to attrition or intentional exclusion and provide justification.	Not Applicable	
For every figure, are **statistical tests** justified as appropriate? Do the data meet the assumptions of the tests (e.g., normal distribution)? Describe any methods used to assess it. Is there an estimate of variation within each group of data? Is the variance similar between the groups that are being statistically compared?	Not Applicable	

Sample definition and in-laboratory replication	Information included in the manuscript?	In which section is the information available? (Reagents and Tools Table, Materials and Methods, Figures, Data Availability Section)
In the figure legends: state number of times the experiment was **replicated** in laboratory.	Not Applicable	
In the figure legends: define whether data describe **technical or biological replicates**.	Not Applicable	

Ethics

Ethics	Information included in the manuscript?	In which section is the information available? (Reagents and Tools Table, Materials and Methods, Figures, Data Availability Section)
Studies involving **human participants**: State details of **authority granting ethics approval** (IRB or equivalent committee(s), provide reference number for approval.	Not Applicable	
Studies involving **human participants**: Include a statement confirming that **informed consent** was obtained from all subjects and that the experiments conformed to the principles set out in the WMA Declaration of Helsinki and the Department of Health and Human Services Belmont Report.	Not Applicable	
Studies involving **human participants:** For publication of **patient photos**, include a statement confirming that consent to publish was obtained.	Not Applicable	
Studies involving experimental **animals**: State details of **authority granting ethics approval** (IRB or equivalent committee(s), provide reference number for approval. Include a statement of compliance with ethical regulations.	Not Applicable	
Studies involving **specimen and field samples:** State if relevant **permits** obtained, provide details of authority approving study; if none were required, explain why.	Not Applicable	

Dual Use Research of Concern (DURC)	Information included in the manuscript?	In which section is the information available? (Reagents and Tools Table, Materials and Methods, Figures, Data Availability Section)
Could your study fall under dual use research restrictions? Please check biosecurity documents and list of **select agents and toxins** (CDC): https://www.selectagents.gov/sat/list.htm	Not Applicable	
If you used a select agent, is the security level of the lab appropriate and reported in the manuscript?	Not Applicable	
If a study is subject to dual use research of concern regulations, is the name of the **authority granting approval and reference number** for the regulatory approval provided in the manuscript?	Not Applicable	

Reporting

The MDAR framework recommends adoption of discipline-specific guidelines, established and endorsed through community initiatives. Journals have their own policy about requiring specific guidelines and recommendations to complement MDAR.

Adherence to community standards	Information included in the manuscript?	In which section is the information available? (Reagents and Tools Table, Materials and Methods, Figures, Data Availability Section)
State if relevant guidelines or checklists (e.g., **ICMJE, MIBBI, ARRIVE, PRISMA**) have been followed or provided.	Not Applicable	
For **tumor marker prognostic studies,** we recommend that you follow the **REMARK** reporting guidelines (see link list at top right). See author guidelines, under 'Reporting Guidelines'. Please confirm you have followed these guidelines.	Not Applicable	
For **phase II and III randomized controlled trials**, please refer to the **CONSORT** flow diagram (see link list at top right) and submit the CONSORT checklist (see link list at top right) with your submission. See author guidelines, under 'Reporting Guidelines'. Please confirm you have submitted this list.	Not Applicable	

Data Availability

Data availability	Information included in the manuscript?	In which section is the information available? (Reagents and Tools Table, Materials and Methods, Figures, Data Availability Section)
Have **primary datasets** been deposited according to the journal's guidelines (see 'Data Deposition' section) and the respective accession numbers provided in the Data Availability Section?	Yes	Acknowledgement
Were **human clinical and genomic datasets** deposited in a public access-controlled repository in accordance to ethical obligations to the patients and to the applicable consent agreement?	Not Applicable	
Are **computational models** that are central and integral to a study available without restrictions in a machine-readable form? Were the relevant accession numbers or links provided?	Not Applicable	
If publicly available data were reused, provide the respective **data citations in the reference list**.	Yes	References and Materials and Methods
